# A cross-sectional network analysis of successful aging in a resilience-based framework

**DOI:** 10.1371/journal.pone.0315445

**Published:** 2025-01-15

**Authors:** Lotte P. Brinkhof, K. Richard Ridderinkhof, Sanne de Wit, Harm J. Krugers, Jaap M. J. Murre

**Affiliations:** 1 Deptartment of Psychology, Faculty of Behavioural and Social Sciences, University of Amsterdam, Amsterdam, Netherlands; 2 Centre for Urban Mental Health, University of Amsterdam, Amsterdam, Netherlands; 3 Amsterdam Brain & Cognition (ABC), University of Amsterdam, Amsterdam, Netherlands; 4 Faculty of Science, Swammerdam Institute for Life Sciences, University of Amsterdam, Amsterdam, Netherlands; Utah State University, UNITED STATES OF AMERICA

## Abstract

Aging inevitably gives rise to many challenges and transitions that can greatly impact our (mental) well-being and quality of life if these are not controlled adequately. Hence, the key to successful aging may not be the absence of these stressors, but the ability to demonstrate resilience against them. The current study set out to explore how resilience and successful aging may intersect by investigating how various resilience capacity-promoting (protective) and resilience capacity-reducing (risk) factors relate to mental well-being and quality of life. Through a large-scale (*N* = 2000, age 55+, 30 factors) network analysis, we established the interplay between risk/protective factors from various domains, including demographics, (mental) health, (environmental) stress, lifestyle, coping/personality, and ageism. We revealed some unique pathways through which each of these factors contribute to individuals’ mental well-being and/or quality of life, and interpreted these findings in terms of a resilience-based framework of successful aging. Our findings emphasize the complexity of factors that can impact quality of life and mental well-being in later life and can steer researchers and practitioners in devising efficacious, multi-pronged interventions that target risk and protective factors simultaneously, thereby maximizing their potential in boosting beneficial outcomes among older individuals.

## Introduction

### The capacity for resilience as crucial determinant of the process that underlies successful aging

As the rapid graying of the world’s population continues, a major challenge is to promote successful aging (Box 1). Successful aging is commonly viewed as a multidimensional concept that encompasses domains of physical, cognitive, psychological and social health [1, 2]. However, retaining high and stable levels of functioning across all domains might be an unattainable goal for the majority of people approaching old age. Many age-related changes negatively affect physical and cognitive functions [3–7], thereby increasing vulnerabilities to chronic diseases (e.g., dementia, osteoarthritis), physical disabilities, and mental health problems (e.g., [8–10]). Moreover, older adults face particular late-life challenges, such as the loss of spouse or loneliness, which further threaten their physical, cognitive, psychological and social health status [11, 12]. Accordingly, it has been argued that there may be different successful aging phenotypes, referring to the diverse combinations of aspects of successful aging in which individuals show more and less favorable trajectories of functioning [13]. As such, the absence of disease, illness or disability is not imperative and older adults may also age successfully despite specific late-life challenges or physical/cognitive limitations, as long as these are well controlled or accepted and therefore have little influence on well-being and quality of life [14–16].

**Box 1**

Over the past decade, various definitions of successful aging have emerged. Initially, the concept primarily focused on extending healthy and functional years in the lifespan, emphasizing the importance of maintaining physical health, cognitive function, and social engagement throughout the aging process. However, more recent definitions have evolved to incorporate subjective aspects of aging and acknowledge individuals’ role = in shaping their own aging process (see e.g., [1, 2, 29, 30]). Increasingly, it is regarded as an aspirational term that accentuates the potential for aging to be a positive and fulfilling experience and focuses on the strength and resources of older adults, rather than on deficits and limitations.

The appropriateness of the term ‘successful aging’ has been subject of an ongoing debate, with many other/related terms being introduced along the way (e.g., healthy aging, vital aging, meaningful aging, productive aging; see e.g., [2]). While each of these terms have their merits, they all emphasize different aspects of aging, and the best term to use may likely depend on the specific context or research goal. For example, if the goal is to emphasize the importance of physical health and well-being in aging, "healthy aging" might be the most appropriate term. If the focus is on maintaining vitality, energy, and engagement in life as people age, "vital aging" might be a better term. "Meaningful aging" may be more appropriate when the goal is to emphasize the importance of finding purpose and meaning in life as we age, while "productive aging" might be used to highlight the contributions that older adults can make to society. We decided to use “successful aging” and define this as the process of developing and maintaining functional ability and competence that enables (mental) well-being and quality of life. This definition also suggests that thriving and flourishing in later life is achievable despite adversity, provided one adequately manages it. Resilience emerges as crucial factor in this regard. Ultimately, what is most important is recognizing the diversity and complexity of the aging process, and to focus on promoting positive aging experiences and outcomes for individuals and society as a whole.

In line with this, we endorse the perspective of the World Health Organization (WHO) and consider successful aging as *the process of developing and maintaining the functional ability and competence that enables (mental) well-being* (i.e., the emotional response to what life is like, reflecting the presence of positive emotions and contentment) *and quality of life* (i.e., the cognitive appraisal of one’s life, in relation to goals, expectations, standards and concerns) *in older age* [15, 17]. This definition places a stronger emphasis on subjective perceptions and importantly, does not imply that increasing vulnerabilities or adversities will inevitably lead to unsuccessful aging. In fact, it recognizes that individuals may thrive and flourish despite such challenges. In our current study, we consider this ‘resilience’ capacity as a crucial determinant of the process that enables mental well-being and quality of life [18–22] (while acknowledging there may be other important capacities or mechanisms involved). Specifically, we posit that a high resilience capacity among older adults may support successful aging by maintaining or regaining mental well-being and quality of life despite the presence of internal (e.g., decline, disease) or external (e.g., loss of spouse) challenges or adversity [23–27].

### The capacity for resilience should be inferred from multiple factors

Resilience is often defined as the process of successfully adapting to difficult or challenging life experiences [28]. However, individuals’ ability to achieve such successful adaptations (i.e., demonstrate resilience) may be influenced by their underlying capacity for resilience. Numerous factors are believed to collectively shape this capacity (e.g., [21]). The current cross-sectional network study aims to provide insights into how resilience and successful aging may intersect by zooming in on this ‘resilience’ capacity. Specifically, we explore *how* various factors that are thought to positively (protective) or negatively (risk) influence individuals’ resilience capacity relate to quality of life and mental well-being. While this design does not allow us to demonstrate causality or address the dynamic interaction between successful aging and resilience, it can highlight how factors that are thought to underlie individuals’ resilience capacity could facilitate successful aging. These insights can serve as a foundation for future longitudinal studies to explore these factors/relationships in greater depth.

Previous research has found that high resilience is associated with numerous positive outcomes, including improved quality of life, (mental) well-being, happiness, life satisfaction, mental health, and the subjective experience of successful aging (e.g., [22, 31–39]). Resilience also appears to support longevity and reduce mortality risk [35, 37, 40–42], as well as encourage desirable lifestyle habits, such as regular physical activity and healthy dietary practices [36, 43–49].

An increasing number of studies has identified factors contributing to high levels of resilience and/or such favorable outcomes. For instance, certain resilience-related trait factors (e.g., the general ability to bounce back from stress), adaptive coping styles, optimism, self-efficacy, self-esteem, social support and involvement, maintaining a physically and mentally active lifestyle, emotional stability, working memory capacity and habit propensity are all thought to contribute to individual differences in the capacity for resilience (e.g., [47, 48, 50–52], and see [21, 53–57] for literature overviews). Additionally, certain personality traits, such as openness to new experiences and conscientiousness, along with socio-demographic and environmental factors, are associated with higher resilience levels [53, 58–61]. While most emphasis has been on investigating the factors that enhance the capacity for resilience, risk factors are also important to consider. For instance, poor mental health (e.g., loneliness, depression), reduced sleep quality, and negative self-perceptions of aging have been linked to lower resilience levels [34, 62–66].

Despite the wealth of studies that have identified factors that contribute to individuals’ capacity for resilience, these factors have often been studied in separate, independent studies. Consequently, little is known about how these factors may interact with one another and (in turn) their relative contributions. However, this is essential to identify the most opportune target points for interventions or personal efforts that could enhance the capacity for resilience and enable individuals to thrive and flourish in their later years. We propose that the field must move beyond capturing the resilience capacity in singular personality characteristics or traits [67], and focus instead on examining the *interplay* between multiple risk (i.e., resilience capacity reducing) and protective (i.e., resilience capacity promoting) factors, and their relative contributions in predicting relevant health outcomes [23, 32, 52, 68, 69], This aligns with the concept of ‘resilience reserve’ ([70]; cf. to cognitive reserve; [71]), which defines resilience as the sum of processes or resources that protect and compensate for challenges or the effects of adversity. Individuals that exhibit a higher overall level of positive resources, are more likely to withstand and overcome challenges (i.e., are more resilient), and therefore show better outcomes in later life.

### Large-scale, cross-sectional network analysis

To take on this challenge, we adopt a large-scale (*N* = 2000, age 55+) network perspective. We explore the combination and interaction of multiple resilience capacity-reducing (risk) and -promoting (protective) factors from various domains and their relationships with mental well-being and quality of life. By doing so, we hope to uncover unique–and perhaps unsuspected–patterns of associations between demographic, environmental, psychological, behavioral, (psycho-)social and cognitive factors, and potential pathways (direct of indirect) through which these factors impact quality of life and mental well-being. This could help to understand how such factors may influence the process that underlies successful aging.

Studying psychological constructs, such as resilience (capacity), by evaluating the complex interplay of relevant factors, has gained increasing popularity in various research fields [72–76]. However, to the best of our knowledge, this is the first study to employ such a comprehensive network approach–including a wide spectrum of both protective and risk factors from different domains (i.e., demographics, (mental) health, (environmental) stress, lifestyle, coping/personality and ageism)–to explore determinants of older individuals’ resilience capacity in relation to relevant outcome variables of interest. While cross-sectional network analysis is a hypothesis-generating technique by nature, this unique and comprehensive network approach enables us to make more validated *predictions* about potential pathways and how individuals may respond to certain internal or external perturbations than would be possible when studying the contribution of factors in isolation. To illustrate, when a certain event or challenge (e.g., loss of friend) interrupts a critical factor (e.g., social support), this may ignite a cascade of symptoms (e.g., loneliness, unhappiness, depression). If these changes are not sufficiently controlled or mitigated by other protective factors in the network (e.g., high self-management or coping abilities), the network may become dominated by dysfunctional connections and, consequently, resilience to perturbation may be lost. This would result in low levels of mental well-being and quality of life.

Such nuanced understanding of the underlying relationships and potential mechanisms that contribute to quality of life and mental well-being in later life can inform future longitudinal studies that aim to study the role of resilience (factors) in predicting the process underlies successful aging more directly. Moreover, it may guide researchers and practitioners in creating effective personalized recommendations or interventions. These may surpass programs that are inspired by the isolated contributions of singular factors and could either target a single risk or protective factor that is likely to have the greatest impact on the entire structure of the network, or multiple factors simultaneously, thereby affecting a number of critical connections from different angles. Thus, by considering the interplay between protective and risk factors, the present study may reveal new insights into how we can promote mental well-being and quality of life among older adults.

## Methods

### Sample characteristics

Data were derived from an ongoing online inventory (in Dutch) on successful aging and resilience (www.seniorendoenmee.nl), covering a multitude of factors that are relevant to successful aging and resilience (approved by the local ethics committee of the University of Amsterdam, 2020-DP-12556). Individuals could register online and had to declare to meet the entry requirements. Although age-related transitions or challenges are typically anticipated to occur at a more advanced age, some individuals may already experience challenges or gradual changes in their social, physical or mental abilities at a younger age. To account for this variability, we set a relatively low minimum age requirement of 55 years, with no maximum age limit. In addition, participants were required to reside in the Netherlands and have no diagnosed form of dementia. Additional exclusion criteria included insufficient Dutch language proficiency, impaired vision, and inability to use a computer or laptop independently (e.g., due to severe symptoms expression of a neurological/neurodegenerative disease such as Parkinson, amyotrophic lateral sclerosis (ALS), multiple sclerosis (MS) or rheumatism or any other physical or mental impairment). After registration, participants received a detailed information brochure via email. After providing their online consent by clicking an acceptance box at the bottom of the agreement, participants could start the inventory (see *Operationalization* for more details).

The total number of participants that completed the entire inventory at the 30th of March (2023) was 2886, of which 176 did not provide a reliable postal code, did not complete the inventory within 14 days or had missing data for one of the variables of interest. Finally, 217 participants were excluded based on their physical activity score. Based on previous experiences with the dataset, showing a considerably skewed distribution for our physical activity measure [32], we subjected the physical activity variable to a robust median absolute deviation outlier detection mechanism (threshold 2.5; [77]). Data of the first 2000 individuals of the 2493 remaining eligible participants were selected for the current study, in conformity with our pre-registered intention. Participants (males:females:other = 674:1325:1) had a mean age of 68.50 (SD 7.06, 55–93) and reported a relatively high educational attainment (i.e., 86% completed a high education level (Verhage 6 or 7; [78]; similar to UNESCO’s ISCED scale; [79]). Noteworthy, these individuals participated in the midst of the COVID-19 pandemic (October 5, 2020 –May 21, 2021).

### Operationalization

The inventory consisted of three parts, delivered online through Qualtrics (www.qualtrics.com) and Neurotask Scripting (scripting.neurotask.com; [80]) and regulated by the LOTUS platform (a webtool developed and managed by the University of Amsterdam). Completion of the inventory took approximately 1 to 1.5 hours in total (20 to 30 minutes per part), but participants were given the flexibility to take short breaks or divide their participation over several days. To minimize framing effects, we presented certain questionnaires (e.g., about depression and anxiety) before psychological or cognitive tests, ensuring that later questions would not be influenced by these.

### Materials

The inventory includes over 200 different factors. For the current study, we considered a total of 28 factors, selected based on their recurrence in previous resilience-related studies and our professional judgment, drawing from personal experiences and observations of relevance in this context. Table 1 lists the different constructs of interest, including mental well-being and quality of life as outcome variables of interest, with their main references includes in the legend. Details on how each construct score has been calculated are included in S1 Appendix. For the continuous variables, the mean, standard deviation and minimum and maximum values are also given in Table 1. Frequency tables of the categorical variables “history of physical and/or neurological diseases” and “history of mental health disorders” are included in S2 Appendix.

**Table 1:** Descriptive statistics of the variables of interest.

Construct (abbreviation; possible range),	M	SD	Min	Max
*Outcomes*				
Mental well-being (MWB; 12–70)^a^	55.6	5.88	34	70
Quality of life (QoL; 24–120)^b^	94.7	9.46	51	120
*Demographics*				
Socio-economic status (SES; 0–100)^c^	85.0	10.3	33.3	100
Urbanisation grade (URB)^d^	2859.0	2665.0	0	15173.2
*Lifestyle*				
Contact level (CON)^e^	168.8	73.9	0	310
Health (HEA; 0–100)^f^	82.8	13.6	9.38	100
Sleep quality (SQ; 0–21)^g^	5.3	3.22	0	19
Physical activity (PHY)^h^	3619.1	2098.2	0	9225
Alcohol use (AU; 0–12)^i^	3.35	2.15	0	11
Prospective and retrospective memory failure (PRM; 8–80)^j^	24.5	4.8	16	55
*(Mental) Health*				
Happiness (HAP; 0–10)^k^	7.80	1.02	2	10
Depression (DEP; 10–40)^l^	15.9	4.59	10	38
Anxiety (ANX; 7–28)^m^	11.0	3.32	7	27
Loneliness (LON; 0–11)^n^	2.76	3.07	0	11
Social-support discrepancy (SSD; 12–36)^o^	15	4.43	12	36
Physical or neurological diseases (PND)^p^	-	-	-	-
Mental health disorders (MHD)^q^	-	-	-	-
Boredom (BOR; 1–5)^r^	1.74	0.74	1	5
*Stress*				
Perceived stress (PS; 0–40)^s^	10.5	5.42	0	32
Major life events (MLE;0–90)^t^	7.13	6.42	0	46
Stringency index (SI; 0–100)^u^	70.2	8.52	0	82.4
*Coping/Personality*				
Ability to bounce back or recover from stress (BRS; 6–30)^v^	21.3	3.88	7	30
Positive appraisal style (PAS)^w^	0.04	0.6	-1.91	1.7
Behavioral coping (BC; 8–32)^x^	21.5	3.66	8	32
General self-efficacy (GSE; 10–40)^y^	32.6	4.14	10	40
Self-esteem (SE; 10–40)^z^	33.0	4.17	16	40
Self-management ability (SMA; 0–100)^aa^	69.4	11.5	30	98.9
*Ageism*				
Negative self-perceptions of aging (SPoA; 21–105)^ab^	51.8	9.18	24	87
**Perceived negative ageism (PNA; 5–25)** ^ac^	7.39	2.61	5	23
**Perceived positive ageism (PPA; 2–15)** ^ac^	9.56	2.28	3	15

Details on how each construct is measured/assessed can be found in S1 Appendix.

^a^ Ikink et al. (2012) [81], Tennant et al. (2007) [82],

^b^ Gobbens and van Assen (2016) [83], Power et al. (2005) [84],

^c^ Composite score: average of the subjective SES, income and education level,

^d^ postal-code inferred measure of the population density, quantified as the average number of addresses per km^2^ within a circle with a radius of 500m from the geographical centre of individuals’ postal code area,

^e^ Composite score: score: the number of days with social contacts weighted by satisfaction with contact and interactions,

^f^ Aaronson et al. (1998) [85], Ware and Sherbourn (1992) [86],

^g^ Buysse et al. (1989) [87],

^h^ Composite score: score: physical activity duration weighted by MET,

^i^ Babor et al. (2001) [88],

^j^ G. Smith et al. (2000) [89]

^k^ Abdel-Khalek (2006) [90],

^l^ Andresen et al. (1994) [91],

^m^ Zigmond and Snaith (1983) [92],

^n^ de Jong-Gierveld and van Tilburg (1999) [93],

^o^ van Sonderen (1993) [94], Kempen and van Eijk (1995) [95], van Eijk et al. (1994) [96],

^p,q^ categorical,

^r^ self-generated single-item question,

^s^ Cohen et al. (1983) [97],

^t^ self-generated items, based on several previous lists,

^u^ https://www.bsg.ox.ac.uk/research/research-projects/coronavirus-government-response-tracker,

^v^ Leontjevas et al. (2014) [98], B. W. Smith et al. (2008) [99],

^w,x^ Veer et al. (2021) [52],

^y^ Schwarzer and Jerusalem (1995) [100], Teeuw et al. (1994) [101],

^z^ Rosenberg (1979) [102], Franck et al. (2008) [103],

^aa^ Cramm et al. (2012) [104],

^ab^ Slotman et al. (2015) [105],

^ac^ Brinkhof, de Wit, et al. (2022) [106].

Originally, to evaluate the history of physical and/or neurological diseases, 13 different categories were included. Based on participants’ responding, some of these categories were combined, resulting in eight categories in total, to ensure sufficient data per category to reliably assess relationships with other variables. A similar procedure was adopted for history of mental health disorders, resulting in three categories.

After encountering severe doubts about the quality of the data related to the two cognitive factors (due to technical difficulties) that were pre-registered to be included in the analysis, the decision was made to exclude them from the analysis to ensure the validity and reliability of the study findings. Instead, based on new insights gained after pre-registration, two other factors were included (i.e., perceived negative ageism and perceived positive ageism), as they were deemed highly relevant predictors of mental well-being and quality of life (e.g., [106, 107]). These factors are highlighted in the table.

### Data analysis

#### Network estimation

The R-packages *mgm* and *bootnet* were used to estimate pairwise (*k* = 2) Mixed Graphical Models at group level [11, 108–110], allowing us to model each relevant variable on its proper domain (i.e., Categorical and Gaussian). Lasso regularization (ℓ_1_) was applied to reduce the inclusion of spurious edges to obtain a sparse network with high specificity [111, 112]. This ℓ_1_-penalty was weighted by a hyper-parameter (ɣ) of 0.25, selected using the conservative Extended Bayesian Information Criterion (EBIC). The edges of the Mixed Graphical Model specify the strength of the association (positive or negative) between two factors (i.e., nodes), irrespective of the other factors of the network (cf. to partial correlations/multiple regression coefficients). Nodes are considered to be conditionally independent is they are not directly connected to one another.

Interactions between variables were visualized using the R package *qgraph* [113], with the thickness and saturation of the edges signifying the magnitude of the relationships. According to the Fruchterman and Reingold algorithm [114], nodes with stronger associations were positioned at the center of the network and nodes with weaker connections were placed in the periphery.

The stability of the edge weights was assessed by using *non-parametric bootstrapping* (using resampled data with replacement, 1000 samples; [115]). Quantile intervals were constructed around the edge weights, with narrow intervals reflecting more stable edge weights.

#### Nodewise predictability

The nodewise predictability (i.e. how well a given node is predicted by all its neighboring nodes in the network: e.g., proportion explained variance; [116]) was computed and visualized in the network using the R packages *mgm* and *qgraph* [110, 113, 117]. To clarify, we aimed to identify to what extent a certain node (e.g., quality of life) could possibly be changed by intervening on the nodes directly associated with it, but also to what extent nodes are not explained by factors included in the network. When the node-wise predictability is low, there might be other variables, which are not included in the network, that interact with this node.

#### Identification of communities within network structure

Since interpreting individual interactions in large networks can be challenging, we further analyzed the structure of the network by identifying strongly connected subgraphs (or communities) in the network [118]. Despite the presence of conceptually defined communities, such as (mental) health, coping/personality, and lifestyle, we were particularly interested in identifying empirically derived communities and the differential roles of the nodes within and between them. That is, psychological constructs with many and strong connections within a certain community could be thought of as the core of a community, whereas factors that belong to multiple communities facilitate the communication between certain domains (see *Local Structure Analysis* below for more information on how the differential roles will be established).

We used the CliquePercolation R-package to run the *clique percolation algorithm* for weighted networks [119–121], and we identified *k*-cliques (i.e., fully connected subgraphs, or communities, with *k* nodes). This method allows for the identification of nodes that belong to multiple communities (i.e., cross-loading), rather than only one. The adjacency matrix of the estimated Mixed Graphical Model was used to optimize *k* (i.e., the smallest clique size, with *k* = 3 as minimum required by the algorithm) and *I* (i.e., intensity threshold; how strong the average relation among nodes within a community needs to be in order to be detected as community) using the *cpThreshold* function (with the CFinder algorithm; for more details see [119, 122]). This function determines the threshold for each *k* and *I* combination and identifies the number of communities and isolated nodes. The optimal *I* for each *k* is just above the point of the emergence of the gigantic component (i.e., with many nodes belonging to one community). An indicator of this point is the *ratio* of the largest to second largest community sizes. *I* and *k* are optimal if the largest community is twice as large as the second largest (i.e., *I* at which the ratio crosses 2; [120, 121]).

However, this point can be unstable in case of a small number of communities. As our network includes 30 nodes, which can be considered moderately large for community analyses, which places us in a boundary area. Therefore, we also explored an alternative approach to optimize *I* and *k*: relying on the *entropy* of the community partition (i.e., the most surprising community partition; [123]). In calculating entropy, isolated nodes are considered as a separate pseudo-community, while shared nodes are divided equally among the communities to which they belong. As such, entropy gives preference to communities that have similar sizes and only a few isolated nodes. The *I* and respective *k* corresponding to the highest entropy was considered optimal. To estimate whether this entropy value is higher than expected by chance, a permutation test was conducted using the *cpPermuteEntropy* function. This function creates 100 permutations of the Mixed Graphical Model extracts the highest entropy for each *k*, and determines a 95% confidence interval of the entropy for each *k*.

Both the ratio and entropy indicator were evaluated. The optimal *k* and *I* were chosen by visually inspecting their respective community networks and choosing the option that corresponds to the community network that *does not* include a giant component to which most nodes belong, but rather has multiple communities of smaller sizes, and has a relatively low number of nodes being isolated from any of the communities.

#### Local structure analysis

To examine the local structure of the communities, we applied a similar approach as described by Blanken and colleagues [122]. The edge weights of the direct relationships that a certain node had within its community were summed and defined as the *stabilizing index*; the edge weights of the direct relationships that a certain node had with nodes of other communities were summed and defined as *communicating index*. These indices provide specific insights into the factors that play a crucial role at the local level (within and between sub-networks), and may serve as opportune target points for interventions.

We also performed some additional exploratory analyses, where we zoomed in on the subscales of some factors, namely quality of life, self-management ability, loneliness, health, self-perceptions of aging. These analyses were performed at the community level, where separate Mixed Graphical Models were estimated for each community that included at least one of the multi-dimensional factors. The subscales or dimensions of the multi-dimensional factor were included as separate nodes. This enabled us to identify how subscales or dimensions relate to other factors within the same community. Importantly, these analyses do not allow us to say anything about the interplay of the subscales with nodes related to other communities. Although generalization to the primary model may seem compelling, the estimated network structure of each community will change as a consequence of the altered set of variables and will therefore not exactly resemble the structure and corresponding edge weights of nodes within and between communities.

#### Moderated network models

Moderated Mixed Graphical Model (or Moderated Network Models) were estimated to determine the role of opportune moderators and assess potential differences across networks of subgroups with favorable and less favorable characteristics (e.g., general self-efficacy; negative self-perception of aging; depression; [124]. Details on this analysis are reported in S1 Appendix. We did not find any moderation effects, which seems most plausibly explained by a lack of statistical power.

## Results

Means and standard deviations of the Gaussian variables of interest are shown in Table 1. As our analyses relied on cross-sectional data, causal inferences cannot be made. However, in order to ensure comprehensive reporting and interpretation of the results, we use causal terms whenever they are deemed plausible. Any ambiguous relationship will be carefully addressed in the discussion.

### Which factors contribute to overall quality of life and mental well-being?

**Fig 1:**
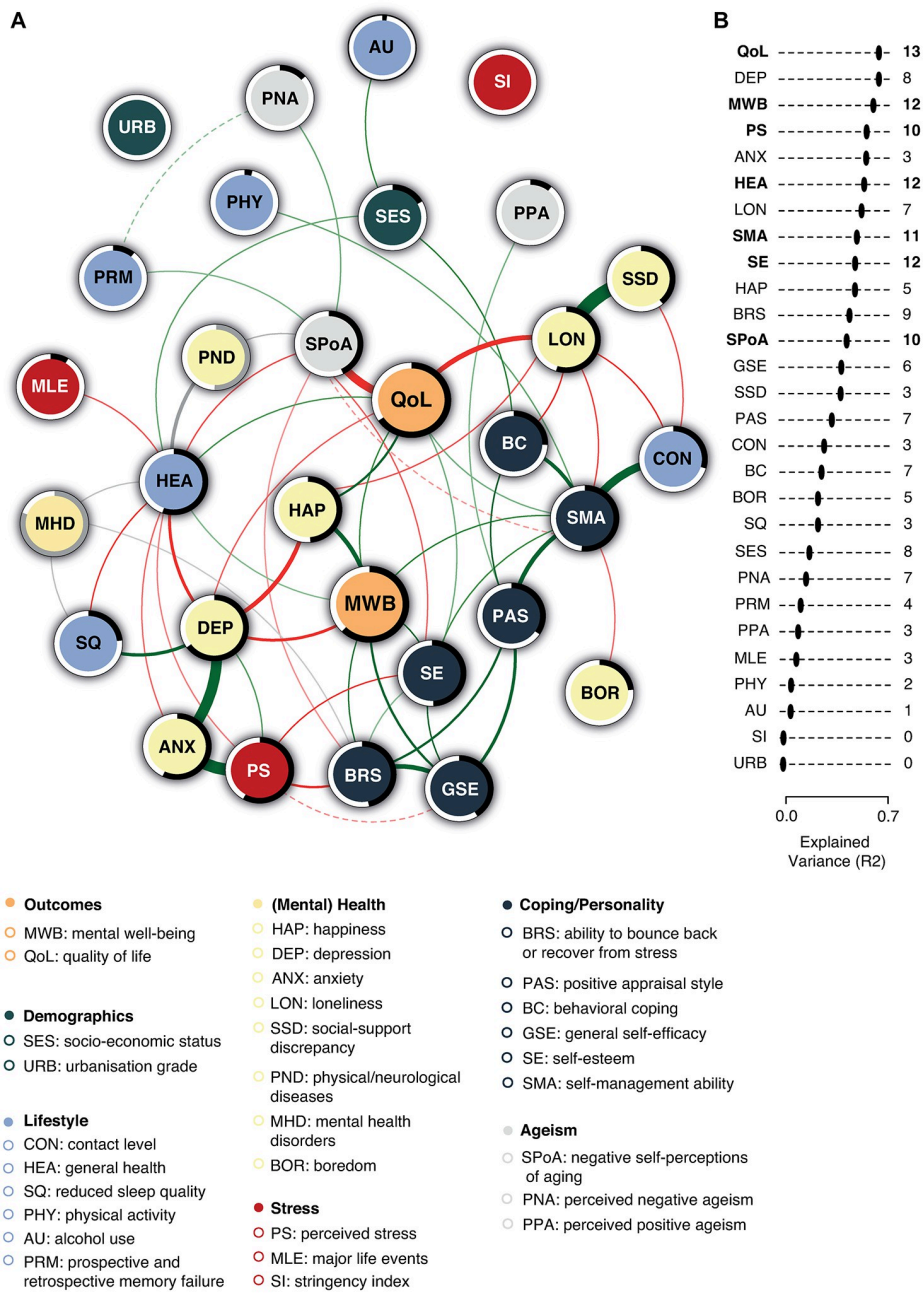
Mixed graphical model. **Panel A:** Green lines (i.e., edges) indicate positive relationships, while red lines indicate negative relationships. Grey edges indicate pairwise interactions involving categorical variables. The exact nature of these interactions are described in text. The width of all lines is proportional to the absolute value of the strength of the relationship. Solid lines depict relationships that were non-zero more than 90% of the bootstrap samples (i.e., very stable), and dashed lines depict relationships that were non-zero between 85% and 90% of the bootstraps (i.e., relatively stable). (Relatively) unstable relationships (non-zero < 85%) are not shown to improve visibility, but can be found in Table 1 of S2 Appendix. The black rings around the Gaussian variables indicate the proportion of variance explained (R^2^, i.e., node-wise predictability) by neighboring nodes. The grey rings around the categorical variables indicate the accuracy. In **panel B**, Gaussian variables are ordered according to their node-wise predictability. The numbers in the right column indicate the number of edges that are connected to each node (including unstable edges that are not shown in the figure). The six nodes that were most central are shown in bold.

The MGM revealed that all nodes were directly or indirectly connected to each other, except for urbanization grade and the COVID-stringency index (Fig 1A). Most variables were related to several other variables, but the most central nodes, which had associations with many other variables, were quality of life, mental well-being, general health, negative self-perceptions of aging, self-management ability, self-esteem, and perceived stress (Fig 1B). Although a direct relationship between mental well-being and quality of life was observed (0.10, cf. to partial correlations), both constructs were especially strongly related through indirect pathways. Below, some interesting relationships relevant for either mental well-being, quality of life, or both will be highlighted. Strengths of all relationships are summarized in Table 1 of S2 Appendix. The node-wise predictability estimates revealed that the variables included in the network accounted for 62% and 66% of the variance in mental well-being and quality of life, respectively. Other node-wise predictabilities are shown in Fig 1B.

When aiming to improve mental well-being directly, self-efficacy seems to be the most opportune target point (0.15). Other malleable coping/personality factors that were directly related to mental well-being include self-esteem (0.13), the subjective ability to bounce back or recover from stress (0.12) and self-management ability (0.11). Mental well-being appeared to be centered around several mental health constructs. Those reporting relatively high mental well-being showed increased happiness (0.19) and reduced depressive symptomatology (-0.16), which was in turn related to less anxiety (0.32) and less perceived stress (0.08; also indirectly via anxiety: 0.34). Interestingly, no direct association between anxiety and mental well-being was observed, indicating that depressive symptomatology plays a crucial mediating role here. Depressive symptomatology also seems to mediate the relationships of mental well-being, happiness, anxiety, and perceived stress on the one hand with reduced sleep quality (0.17) and general health (-0.16) on the other. The malleable capacities directly related to the cluster of mental health constructs were self-esteem (-0.13), the ability to bounce back and recover from stress (-0.15) and self-efficacy (-0.06; although less robust). These were all directly related to perceived stress. This suggests that self-esteem, one’s subjective ability to bounce back or recover from stress and/or self-efficacy are the most opportune target points for interventions that aim to reduce perceived stress, which may indirectly alleviate anxiety, and depressive symptomatology, and thereby improve sleep quality and/or general health. Indeed, this may then also improve mental well-being.

Quality of life also seemed to be directly influenced by self-esteem (0.06) and self-management ability (0.06), with the latter also exerting its influence through its favorable impact on loneliness (-0.20; 0.10), self-esteem (0.10; 0.06) and mental well-being (0.11; 0.10). Although behavioral coping did not seem to strongly contribute to mental well-being, its association with loneliness (-0.13) and self-management ability (0.18) suggests adequate behavioral coping can promote quality of life indirectly. Loneliness was also strongly associated with higher social-support discrepancy (0.38) and intervening on that factor may also trigger a cascade of positive changes in favor of older adults’ quality of life. Besides these influences through the malleable coping/personality related factors, quality of life seemed to be particularly strongly affected by individuals’ self-perceptions of aging (-0.27). More negative self-perceptions of aging were also associated with lower self-esteem (-0.08), general health (-0.09), the subjective ability to bounce back or recover from stress (-0.05), self-management ability (-0.05), as well as increased prospective and retrospective memory failure (0.06). Interestingly, especially those who reported high levels of perceived negative ageism (0.07) and those with (a history of) cancer or neurological/cardiovascular problems seemed to have more negative self-perceptions of aging (see details on categorical findings in S2 Appendix, Table 2), suggesting these might be important risk factors. Both having (a history of) cancer or neurological problems also seem to negatively impact individuals’ general health reports, along with other physical/neurological problems, such as diabetes mellitus, dizziness, rheumatic disorders and the consequences of a heart attack/cardiac arrest or brain trauma. Other relationships with categorical variables are described in Table 3 of S2 Appendix.

#### Communities within network structure

**Fig 2:**
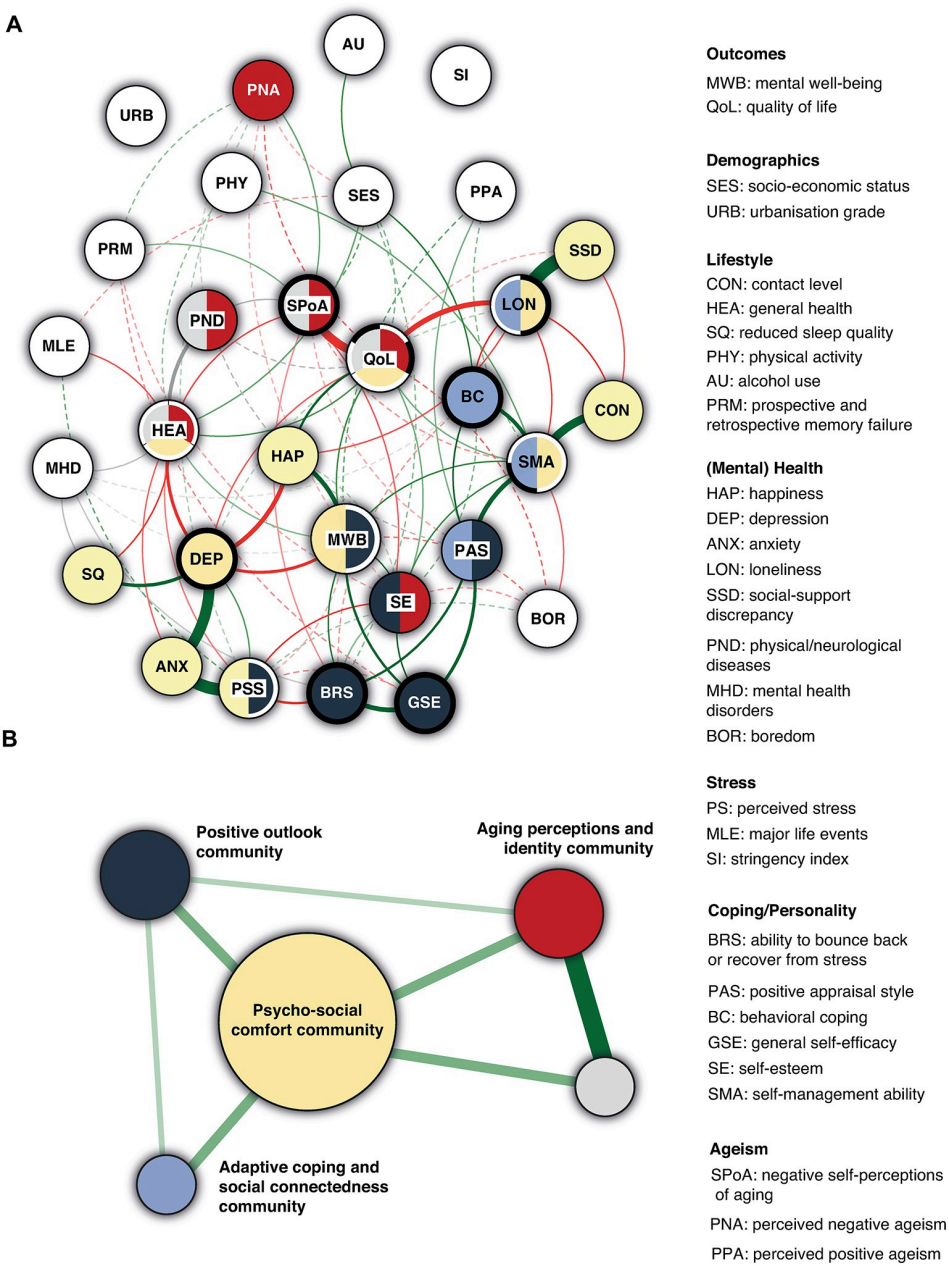
Community structures. **Panel A:** Green lines (i.e., edges) indicate positive relationships, while red lines indicate negative relationships. Grey edges indicate pairwise interactions involving categorical variables. The exact nature of these interactions are described in text. The width of all lines is proportional to the absolute value of the strength of the relationship. Solid lines depict relationships that were non-zero more than 90% of the bootstrap samples (i.e., very stable), and dashed lines depict relationships that were non-zero less than 90% of the bootstraps (i.e., unstable). Colors of the nodes depict the community they belong to. Within each community, the two or three nodes with the highest stabilizing index have a black ring, whereas the two nodes with the highest communicating index have a white ring. This represents an arbitrary selection criterion, devoid of any theoretical assumptions, solely intended to accentuate the most influential nodes. Note that some nodes rank high both as communicator and stabilizer, either across communities, or within one community. A total of 9 notes was isolated. In **panel B**, the associations between communities is shown, with the width of the nodes being proportional to the number of nodes included in that community.

Relying on the ratio of the largest to second largest community *or* the entropy indicator pointed towards different *k* and *I* values as optimum (ratio: *k* = 3, *I* = 0.110; entropy: *k* = 4, *I* = 0.080). The ratio indicator led to a community network of five components, with relatively small sizes (*n* = 12, 6, 6, 4 and 4); 20 (out of 30) nodes corresponded to at least one community (i.e., 10 nodes were isolated; Fig 2A). All communities were directly or indirectly associated with one another (Fig 2B). When relying on the entropy indicator, a total of five (relatively similar) communities was found, but 13 nodes were isolated. This was considered suboptimal in comparison; hence we deemed the community network based on the ratio indicator to be optimal.

#### Local community analyses

Several factors loaded on only a single community, whereas other variables were part of two or three communities (see Fig 2A and Table 2). The **yellow** community is characterized by its comprehensiveness, comprising both mental well-being and quality of life and a cluster of mental health constructs, as well as several social factors (i.e., loneliness, social support discrepancy, and contact level). Accordingly, this community is hereafter referred to as the **psycho-social comfort community** (note that the labels we assigned to the communities are merely convenient designations, so that beyond their immediate denotation, the terms represent a connotative tenor rather than a distinct identity). In this community, depression and loneliness showed the highest stabilizing index; quality of life and self-management ability showed the highest communicating index.

**Table 2:** Stabilizing (S) and communicating (C) index for each node, within its corresponding community/communities.

	Psycho-social comfort		Aging perceptions and identity				Adaptive coping		Positive outlook	
	YELLOW		RED		GREY		BLUE		NAVY	
	*S*	*C*	*S*	*C*	*S*	*C*	*S*	*C*	*S*	*C*
HAP	0.61									
MWB	0.64	0.42							0.42	**0.64**
DEP	**1.19**									
QoL	0.71	**0.59**	**0.59**	**1.07**	**0.44**	**1.21**				
ANX	0.74									
BRS									**0.66**	
PAS							0.32	0.31	0.31	0.32
BC							**0.44**			
PS	0.52	0.37							0.37	**0.52**
GSE									**0.69**	
SE			0.18	0.44					0.44	
SMA	0.54	**0.47**					**0.47**	**0.54**		
LON	**0.93**	0.23					0.23	**0.94**		
SSD	0.50									
HEA	0.57	0.36	0.36	**0.83**	0.36	**0.83**				
PND			0.41		0.36	0.41				
SQ	0.29									
PNA			0.23							
CON	0.48									
SPoA			**0.61**		**0.45**	0.61				

The nodes with the highest stabilizing index can be expected to be most influenced by their community member or have a strong impact on nodes within its community. The nodes with the highest communicating index can be expected to play an important role in connecting different communities (i.e., ‘bridge constructs’). Bold faced value correspond to the two or three (in case of the yellow community, because SMA and HEA have relatively similar communicating indices and MWB and LONE have relatively similar stabilizing indices) nodes with the highest stabilizing and communicating index of each community. This represents an arbitrary selection criterion, devoid of any theoretical assumptions, solely intended to accentuate the most influential nodes.

The **navy** community includes the subjective ability to bounce back or recover from stress and general self-efficacy as strong stabilizers, and mental well-being and perceived stress as communicators. The other constructs included are self-esteem and positive appraisal style. This six-factor community will be referred to as the **positive outlook community**, emphasizing the benefit of having a positive mindset: believing in one’s own abilities and approaching challenges and situations with optimism.

The **blue** community will be referred to as the **adaptive coping and social connectedness community** and includes four constructs: loneliness, self-management ability, positive appraisal style and behavioral coping. This community reflects the importance and comprehensive nature of adaptive coping, encompassing both individual-level strategies and the broader social context in which coping occurs. In this community, self-management ability ranks high as stabilizer and communicator. Additionally, behavioral coping is a strong stabilizer and loneliness a strong communicator, with many connections with the **psycho-social community**.

Interestingly, the **red** community encompasses the entire **grey** community, suggesting that while the red community is a strongly connected subgraph (i.e., surviving the threshold *I*), the variables corresponding to the grey community are even more densely connected. Across red and grey communities, similar nodes were ranked as strongest stabilizers (i.e., quality of life and self-perceptions of aging) and communicators (i.e., quality of life and health). To avoid unnecessary repetition, only the largest community (red) will be evaluated. In addition to quality of life, self-perceptions of aging and health, the red community includes self-esteem, perceived negative ageism and physical and neurological diseases. This community will be referred to as the **aging perceptions and identity** community and emphasizes the importance of positive self-perceptions (about aging, but also in relation to self-esteem), and how aging individuals need to effectively manage ageism and their health, and ultimately construct their identities in the context of aging.

#### Exploratory local structure analysis

**Fig 3:**
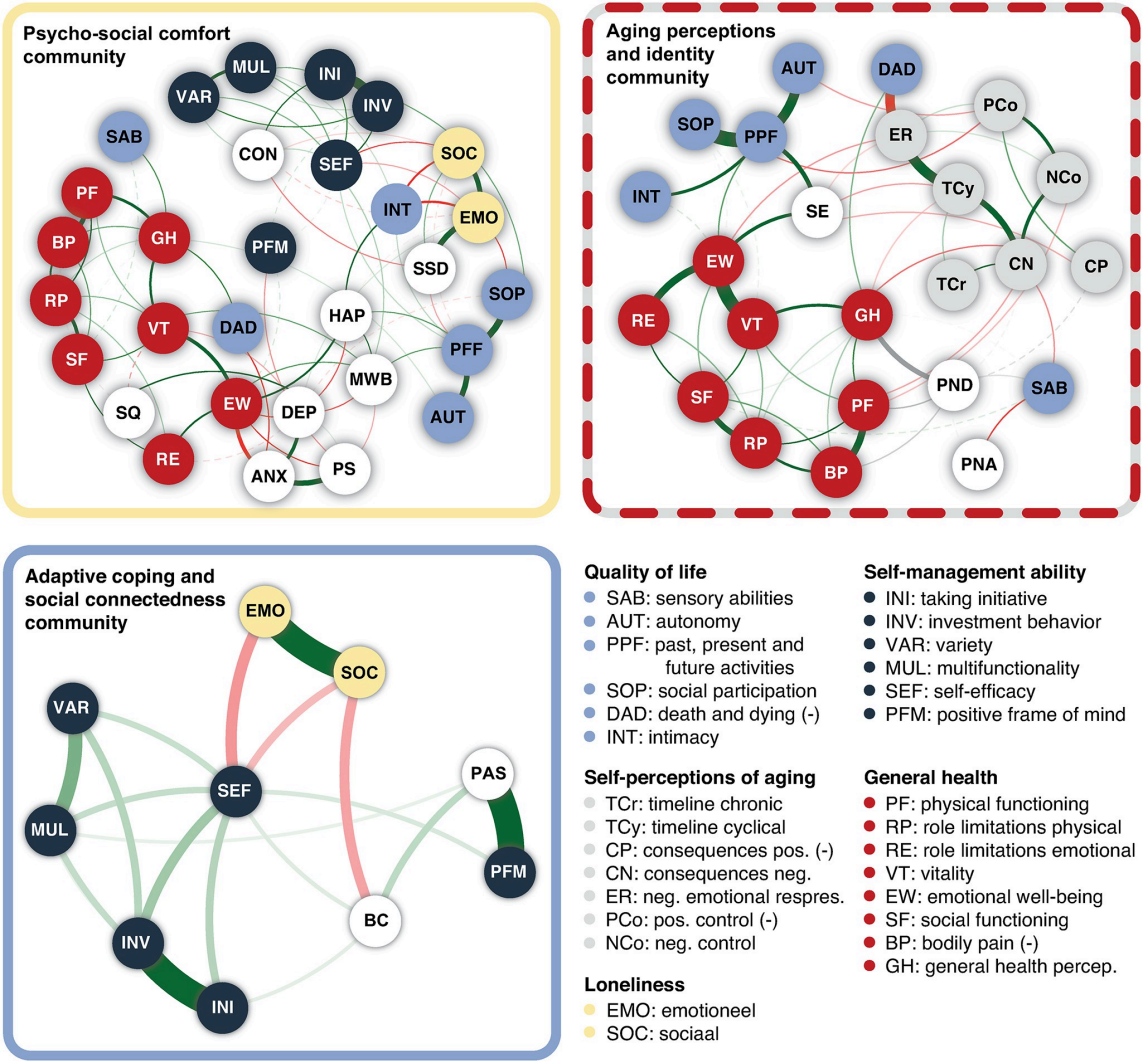
Exploratory local structure analysis with multi-dimensional constructs being split according to their subscale. Green lines (i.e., edges) indicate positive relationships, while red lines indicate negative relationships. Grey edges indicate pairwise interactions involving categorical variables. The width of all lines is proportional to the absolute value of the strength of the relationship. Solid lines depict relationships that were non-zero more than 90% of the bootstrap samples (i.e., very stable), and dashed lines depict relationships that were non-zero less than 90% of the bootstraps (i.e., unstable). Given that variables within the same community should already be strongly related to one another, only relationships stronger than 0.05 are shown. ANX (anxiety), DEP (depression), PS (perceived stress), MWB (mental well-being), HAP (happiness), BRS (the ability to bounce back and recover from stress), GSE (general self-efficacy), SE (self-esteem), SQ (worse sleep quality), CON (contact level), SSD (social support-discrepancy), SES (socio-economic status), PND (physical/neurological diseases), PNA (perceived negative ageism), PAS (positive appraisal style), BC (behavioral coping) are not split into any subscales and shown in white. The constructs for which subscales were considered are color-coded. The grey community is only evaluated as part of the red (aging perceptions and identity) community. The navy (positive outlook) community is not included, as it does not include any constructs with subscales.

Fig 3 illustrates the local communities, with multi-dimensional constructs being split according to their subscale. Again, since the grey community is embedded within the red (**aging perceptions and identity**) community, only the red community was evaluated. The **positive outlook** community did not include any construct that consists of different subscales, and was therefore not considered in this exploratory analysis. The most surprising/interesting relationships will be highlighted in text below; the strengths and bootstrapping results of all relationships are summarized in S2 Appendix (Tables 4–7). The relationships with categorical variables are also described in S2 Appendix (Table 8).

*Psycho-social comfort (yellow) community*. Evaluating the local structure of the psycho-social comfort community, we found that mental well-being was directly related to the SF-36 subscales emotional well-being (EW) and vitality (VT). In addition, some interesting inter-relationships were found among the SF-36 and WHOQOL-OLD subscales. Worries and fears about dead and dying (DAD) was directly related to worsened general health perception (GH) and indirectly (via anxiety) to emotional well-being (EW). Sensory abilities (SAB) and autonomy (AUT) were also directly associated with better GH, as well as social functioning (SF) in case of SAB. The relationship between quality of life and mental well-being seemed to be driven mainly by the association with the satisfaction with past, present and future (PPF) activities, either directly, or through the effects of happiness. This suggests that those having better mental well-being are happier and may therefore be more satisfied about past, present and future activities. Alternatively, it may indicate that individuals who are more satisfied with PPF activities are happier, and therefore experience better mental well-being.

Of all SMAS subscales, self-efficacy (SEF) was most strongly associated with both emotional (EMO) and social (SOC) loneliness, and thereby indirectly with the intimacy (INT) and social participation (SOP) subscales of the WHOQOL-OLD. SEF was also directly associated with higher satisfaction with PPF activities. Interestingly, intimacy was affected by both subtypes of the loneliness construct.

*Aging perceptions and identity (red) community*. In addition to previously reported associations between subscales of the WHOQOL-OLD and SF-36, the local structure of the aging perceptions and identity community showed some interesting interrelations with subscales of the self-perceptions of aging construct. Most prominently, those with more worries and fears about dead and dying, also reported more negative emotional responses to aging (e.g., depressed when thinking about getting older). More negative emotional responses were, in turn, associated with reduced emotional well-being. Those with a worsened general health (subscale SF-36) reported a stronger cyclical awareness of one’s age (e.g., going through phases of feeling old) and more negative beliefs about the impact of aging. Those who had more negative beliefs about the impact of aging and how much control they have over certain aspects of aging, reported reduced physical functioning. Finally, individuals with few positive beliefs about the extent to which one has control over various aspects of aging, were less satisfied with their past, present and future activities and experienced less autonomy.

Self-esteem also played an interesting, potentially buffering, role. Those scoring high on self-esteem reported fewer emotional responses to aging and had less negative emotional responses, more positive beliefs about the impact of aging (e.g., becoming wiser), and were less cyclically aware of their age.

*Adaptive coping and social connectedness (blue) community*. In addition to the crucial role of self-efficacy in linking self-management ability to loneliness, we found that behavioral coping was strongly linked to social loneliness (and thereby only indirectly to emotional loneliness). As expected, a strong positive appraisal style co-occurred with a positive frame of mind but was only indirectly related to emotional and social loneliness through behavioral coping and the self-management ability subscales.

## Discussion

In later life, a strong capacity for resilience may support the process that underlies successful aging by preserving or regaining mental well-being and quality of life in the face of internal and external challenges. Through large-scale network analysis (N = 2000, 30 factors), the present study set out to unravel unique and perhaps unsuspected pathways through which various resilience capacity-promoting (protective) and resilience capacity-reducing (risk) factors–pertaining to demographics, physical and mental health, (environmental) stress, lifestyle, coping/personality, and ageism–could contribute to the mental well-being and quality of life of adults aged 55 or older. In this way, we aimed to provide new insights into which and how factors may contribute to older adults’ capacity for resilience, and thereby influence the process that underlies successful aging.

Our findings suggest that mental well-being is strongly associated with various coping- and personality related factors. Self-efficacy, self-esteem, self-management ability, the subjective ability to bounce back or recover from stress, and positive appraisal style all showed beneficial relationships with mental well-being, either directly and/or indirectly through their impact on a cluster of perceived stress, depression and anxiety. These factors also appear crucial for quality of life, with self-management ability and self-esteem exhibiting direct associations, and the other factors having indirect associations. Moreover, quality of life was strongly linked to a cluster of several social factors, including loneliness, social support discrepancy and contact level. Collectively, these patterns of associations form a strongly connected **psycho-social comfort** community, emphasizing the importance of nurturing both mental health and social connectedness and satisfaction, and a smaller, partially overlapping, **positive outlook community**, stressing the benefit of having a positive mindset. A small cluster of loneliness, behavioral coping, self-management ability and positive appraisal style formed the **adaptive coping and social connectedness** community, further emphasizing the advantages of adaptive coping within a social context. In addition, we found a robust link between lower quality of life and negative self-perceptions of aging, which was the most characteristic relationship of the **aging perceptions and identity** community. Health also emerged as a central factor in the network, with widespread connections across multiple domains, emphasizing its relevance in both the psycho-social comfort and aging perceptions and identity community. These findings can inform future longitudinal studies that aim to examine the dynamic relationship between resilience and successful aging more directly. In particular, these insights may help identify potential targets for personalized recommendations or interventions to boost the resilience capacity among older adults, though the effectiveness of such efforts remains to be tested.

To provide a comprehensive understanding, the following sections will delve into the most intriguing observed patterns of associations, discussing them in relation to the network communities that we uncovered. Subsequently, we contextualize these patterns within the broader framework of resilience and successful aging. Next, we will ponder opportune interventions, inspired by present findings, before discussing some general and specific limitations of our approach and outcomes. The discussion closes with a summary and conclusions.

### Mental well-being and quality of life

Our findings support the distinct yet interdependent nature of mental well-being (i.e., emotional response to what life is like, reflecting the presence of positive emotions and contentment) and quality of life (i.e., the cognitive appraisal of one’s life; [17]). Although a direct relationship between both constructs was observed, they were especially strongly related through various indirect pathways. Both mental well-being and quality of life were associated with various similar protective and risk factors. It is evident, however, that certain factors hold greater significance for one construct over the other. This was also confirmed by their differential contribution to specific data-driven communities. In line with their varying emphasis and domains, we found that factors that are known to influence emotions, such as mental health or happiness, were most strongly linked to mental well-being, whereas factors such as self-perceptions of aging and loneliness, which inherently involve internal contemplation and evaluation of one’s beliefs, attitudes and experiences, appeared to be more strongly related to quality of life.

Insights into these contributions can aid the development of more effective and targeted interventions, focusing either on mental well-being or quality of life as the primary outcome variable. The subsequent discussion will explore several opportune interventions. Importantly, it should be recognized that such focused interventions are likely to have positive implications for the other concept (either mental well-being or quality of life) as well, due to the complex interplay and indirect pathways bridging these two concepts. This interplay may subsequently initiate a mutually reinforcing loop, emphasizing that the potential benefits on either outcome variable cannot be considered in isolation.

### Mental well-being: The emotional response to what life is like

In line with the perspective that mental health is one of the preconditions of pursuing mental well-being [125, 126], our network analysis revealed that a cluster of various mental health constructs, notably anxiety, depression, and perceived stress [127–129], was strongly linked to individuals’ happiness and (in turn) to their mental well-being. Whereas depression seemed to have an important role in linking anxiety and perceived stress to both happiness and mental well-being, the level of perceived stress appeared to be the most opportune target point for interventions, due to its strong link to various personality- and coping-related factors (i.e., strong communicator of the positive outlook community). Indeed, lowering perceived stress may help to *regulate* the mutually and reciprocally reinforcing feedback loops between perceived stress, anxiety and depression, in favor of mental well-being [130]. Perceived stress may be reduced by lowering the level of stressor exposure, or by enhancing coping efficiency to mitigate the threat posed by the stressors. In fact, a comprehensive psycho-social resilience intervention, focusing on promoting positive emotions, savoring experiences, using adaptive coping skills, and discussing the impact of ageism or strategies to improve self-perceptions of aging, has been shown to be effective in reducing perceived stress among older adults, even though the program did not directly reduce the number of stressors in the participants’ environment [131, 132]. Among various other exercises, this multi-pronged intervention included activities to build self-esteem and self-efficacy, as well as some Cognitive Behavioral Therapy-informed thought-challenging skills to enhance positive self-perceptions of aging. Our results suggest that self-esteem may be among the main drives behind the effectiveness of this interventions, having strong direct and indirect (through self-efficacy and the subjective ability of bouncing back or recovering from stress) associations with perceived stress. Having more positive self-perceptions of aging may confer more indirect benefits, for instance by enhancing one’s self-esteem [133].

The psycho-social comfort community also included some patterns of association that concur with previously established interplays among perceived stress, depression and anxiety, on the one hand, and sleep quality and health on the other hand (e.g., [134–139]). Again, depressive symptomatology seemed to play a crucial role in linking all of these constructs. The local community analysis revealed that the SF-36 subscales of emotional well-being and vitality, referring to a state of being lively and energetic, played an important role in linking health to mental well-being, both directly and through their relationship with depression. Indeed, vitality has previously been argued to emerge as a component of mental well-being [140], and fostering vitality may contribute to improved emotional appraisal of one’s life.

### Quality of life: The cognitive appraisal of one’s life

#### Psycho-social comfort, positive outlook and social connectedness

Aside from fostering mental health, the identification of the psycho-social comfort community has also suggested the significance of promoting social satisfaction and connectedness. In accordance with previous studies (e.g., [34, 141, 142]), quality of life was strongly linked to loneliness, which in turn had a strong direct association with social-support discrepancy [94]. Interestingly, one’s self-management ability seems to be an important predictor for one’s contact level, as well as decreased feelings of loneliness, both directly and through its positive effects on contact level. This corroborates the idea that the ability to *manage* external resources (e.g., friends, social support) is equally important as *having* such resources for preventing feelings of loneliness [143, 144], and that loneliness is an important mediator of the relationship between contact level and self-management ability on the one hand, and quality of life on the other hand. This introduces an interesting nuance to previously reported findings that were based on a subset of the present study’s data, showing a strong direct association between one’s self-management ability and quality of life [32]. Our local community analysis further reveals that self-efficacious individuals with relatively positive beliefs in their personal competence to achieve important life goals appear less lonely, both socially and emotionally. This concurs well with an earlier study of Nieboer and colleagues [144] and provides further evidence for the idea that maintaining strong self-efficacy beliefs is important to prevent feelings of loneliness, and (in turn) maintain adequate quality of life.

In contrast with previously reported findings [32], a direct link between positive appraisal style (PAS) and quality of life was no longer present in the current extended network. Instead, PAS served an important role in the adaptive coping and social connectedness, and in the positive outlook community. It appears to provide its benefits for quality of life through its positive impact on various other personality- and coping-related factors. These included self-management ability and self-esteem, via general self-efficacy. This aligns well with earlier studies showing that positive reappraisal was associated with higher levels of self-efficacy [145] and self-esteem [146], and with more recent findings of Diotaiuti and colleages [147] that highlight the benefit of using adaptive or functional cognitive appraisal strategies during the COVID-19 pandemic for bolstering individuals’ level of general self-efficacy. Interestingly, these observed patterns contradict the Positive Appraisal Style Theory of Resilience (PASTOR), which states that having the tendency to appraise potentially threatening situations in a positive way is a more proximal cause for resilience, mediating the relationship with other resilience factors and resilience [148, 149]. The present findings suggest that there may be a more complicated interplay between the factors involved, with positive appraisal style also serving more distal functions, that, together, contribute positively to one’s resilience capacity.

Self-management ability still served as important mediator for the association between behavioral coping (adaptive coping and social connectedness community) and quality of life (psycho-social comfort community), but behavioral coping also had a direct negative association with loneliness. We hypothesize that using behavioral coping strategies may help to reduce feelings of loneliness. Our exploratory analyses suggest this may be the case for social loneliness, which may then indirectly reduce emotional loneliness as well. Nevertheless, the behavioral coping scale includes many items that reflect socially supported coping (i.e., requiring the presence or active involvement of others). Hence, this negative relationship may also indicate that those who are feeling lonely and miss people around them, and thus lack social connectedness, are less likely to deploy behavioral coping, signifies the need for other resources to manage feelings of loneliness.

#### Aging perceptions and identity

In addition to these patterns of associations, lower levels of quality of life were also strongly linked to more negative self-perceptions of aging. This is in line with a large number of previous studies [150]. The current study also shows that the association between both constructs is driven by multiple associations among the subdimensions. Previous studies have emphasized that negative self-perceptions of aging can stem from various factors (e.g., [151, 152]), either distal (e.g., biological health, personality or coping abilities) or more proximal (e.g., experiences of age stereotypes). The present study suggests an important role for low levels of self-esteem or the subjective ability of bouncing back or recovering from stress, higher levels of perceived negative ageism, worse memory and health, or having (had) cancer or a neurological or cardiovascular disease. Yet, negative self-perceptions of aging may also act as a risk factor for further deterioration of the same variables (e.g., [153–156]), forming a self-perpetuating feedback loop.

While such a detrimental cycle might be challenging to break, efforts to improve self-perceptions of aging are gaining momentum as recent interventions have shown promising effects [157, 158]. In addition, preventing high levels of the aforementioned risk factors may also help to reduce negative self-perceptions of aging. For instance, campaigns or interventions to reduce ageism among the general population may contribute to lower levels of perceived negative ageism [159] and thereby indirectly to more positive self-perceptions of aging. Efforts to maintain self-esteem in later life may yield similar benefits [133]. Given the diverse combination of risk factors, it seems most promising to target multiple factors at once, potentially igniting a cascade of changes that will then lead to more positive self-perceptions of aging. In turn, this may also help to reduce worries and fears about death and dying, as suggested by our exploratory analyses.

### A broader perspective on successful aging and resilience

Flourishing in later life involves successfully navigating the challenges and adversities associated with aging. Our findings suggest that the effectiveness of this navigation relies on a balance between a broad spectrum of resilience capacity-reducing (risk) and -promoting (protective) factors. In our cross-sectional design, we found that protective and risk factors interact with one another and seem to be associated with quality of life and mental well-being, either through direct or more indirect pathways. Moreover, the association patterns suggest that a change in one factor may trigger a cascade of changes in others. It also suggests, however that while increases in specific risk factors (e.g., perceived stress) may elevate the likelihood of more adverse levels of another risk factor (e.g., depression), such negative effects may be counteracted by high levels of protective factors such as self-esteem. We propose that interrelations between both protective and risk factors (with opposing effects) align with the notion that older adults may age successfully despite limitations/risk factors, as long as these are well controlled or accepted [14–16] and that there may be different pathways to successful aging [13]. Future research incorporating individual differences in adversity and limitations might benefit from considering this idea, and specific dynamics involved(also see ‘*Studying the general capacity for resilience in later life’*). Our findings also support the idea that resilience is difficult to capture in one single variable, and should be studied by examining the interplay between multiple relevant factors. Finally, our findings underscore the importance of bolstering various personality-and coping-related factors, as well as more positive self-perceptions of aging to enhance resilience and promote positive adaptation in challenging circumstances in later life.

Current findings also suggest that, in addition to objective characteristics (e.g., health, contact level), one’s subjective experiences (e.g., happiness, self-perceptions of aging, loneliness) are important for predicting quality of life and mental well-being. This appears to confirm the multifaceted and subjective nature of successful aging [1, 2]. As we see it, each individual has their own unique set of aspirations, goals, and circumstances, which likely shapes one’s perception of what is means to age successfully, and (in turn) the cognitive and emotional appraisal of one’s life. Depending on these diverse life perspectives, and the challenges and transitions one experiences, the specific resources that are necessary to maintain resilience throughout life–and should preferably be stored in one’s *resilience reserve*–may also differ. It seems likely that such different resilience ‘profiles’–in combination with variation in experienced stressors–contribute to distinct successful aging phenotypes (Kok et al., 2017). One person may possess the ability to effectively nurture a positive mindset, while someone else may excel at cultivating a variety of friends. Similarly, one person may demonstrate remarkable adaptability to changes and resilience in the face of personal losses, whereas another person may display a great aptitude for embracing physical limitations. We speculate that such differences can give rise to divergent aging trajectories, but still manifest successful outcomes. Overall, in order to age successfully, individuals may need to possess the capacity to navigate their lives while embracing and leveraging their strengths, as well as recognizing and potentially compensating for their weaknesses (e.g., with additional efforts, inviting support, or by engaging in targeted intervention programs). In other words, we argue that successful aging likely encompasses an individualized process wherein individuals harness their personal resources to maintain resilience in a manner that is uniquely suited to them. This requires a certain level of self-awareness and an understanding of one’s own capabilities, which may improve with accumulated life experiences. This view aligns with many studies that indicate resilience often increases with age [35, 69, 160, 161].

### Using network analyses of resilience to direct and shape interventions?

The comprehensive set of variables incorporated in the present network accounted for a substantial amount of the variance in mental well-being (62%) and quality of life (66%), with numerous direct associations observed for both variables of interest. This suggests the viability of intervening on neighboring nodes or connections. However, as previously discussed in the context of interventions that target perceived stress or self-perceptions of aging, a multi-pronged approach that targets multiple nodes or connections simultaneously is likely the most advantageous. Moreover, given the individual variations in successful aging (trajectories) and the specific areas where additional support to maintain a strong resilience capacity may be needed, it is essential to devise interventions and strategies that cater to the unique needs and circumstances of individuals.

To illustrate this further, let us consider volunteering activities as an example. Volunteering has previously been associated with considerable benefits for individuals’ quality of life and mental well-being [162–164]. By providing opportunities for social interaction, volunteering can increase social support, engagement, and diminish feelings of loneliness and depression (e.g., [165–168]). Moreover, volunteering can offer opportunities for personal growth, skill development or maintenance, and instill a sense of purpose or fulfillment [169–171], thereby contributing to improvements in, for instance, general self-efficacy, self-esteem, and self-perceptions of aging [165, 166, 172, 173]. Finally, volunteering has been linked to a greater capacity to bounce back or recover from stress [174]. Hence, in essence, volunteering has the potential to scaffold many critical connections in the network and therefore bolster one’s overall capacity for resilience from different angles. Moreover, volunteering offers a range of options and opportunities to leverage individual strengths and boost weaknesses, and thereby provides a versatile platform that can cater to individual needs and circumstances, allowing each person to derive specific benefits that contribute to their personal resilience reserve. In contrast, the relatively limited benefits of cognitive training may be attributed to its narrow focus on just a few factors, such as memory and, perhaps, self-perceptions of aging [175, 176], which may only benefit individuals with specific needs. Thus, interventions or programs that can promote the resilience capacity from different angles and provides room for personal nuances are expected to yield greater benefits. Other multi-pronged interventions that could have similar widespread benefits include meta-cognitive planning interventions [177] or the previously discussed psycho-social resilience intervention of Jeste and colleagues [132].

To assess the extent to which personalized multi-pronged interventions, such as engaging in volunteering activities, indeed confer their benefits by scaffolding multiple critical connections within such a comprehensive network, the utilization of Network Intervention Analysis [178, 179] can be a valuable next step. This approach can aid in understanding and optimizing the effectiveness of interventions. Nonetheless, it is important not to disregard uni-pronged interventions entirely, as they still can hold relevance for specific objectives such as utilizing cognitive reappraisal to moderate the relationship between perceived stress and anxiety; [180]. It may also be more feasible and less burdensome to individuals [181], which in turn can lead to better compliance. However, such interventions seem less likely to strongly enhance the overall capacity for resilience.

### Studying the general capacity for resilience in later life

There are two aspects of our approach to studying resilience (capacity) that need to be considered further. First, it is important to acknowledge that while we have selected quality of life and mental well-being as outcome variables of interest, other factors may be relevant as well. We have chosen to focus on these variables because they are crucial to our definition of successful aging, and effectively capture the subjective nature of this process. However, if one would be particularly interested in the factors that could mitigate the risk for depression in later life, depression would likely be the most appropriate outcome variable to focus on. Second, it is imperative to acknowledge that our approach does not account for individual differences in the magnitude of stress or burden experienced by individuals as they navigate through their lives. Other authors (e.g., [182, 183]) rightly emphasized the significance of stressor exposure–ranging from daily stressors to major life events–in understanding an individual’s capacity for resilience. It enables the differentiation between individuals who are highly reactive to stressors, as evidenced by a negative (mental) health status despite a limited number of stressors, and those who appear more resilient, maintaining a positive (mental) health status despite being exposed to numerous stressors. Although we recognize the value of this approach when studying an individuals’ capacity for resilience in the face of one or more specific late-life challenges (e.g., retirement, loss of a beloved one), it appears less suitable for our objective of exploring the factors that predict individual variations in how well people navigate or adapt to late-life challenges in general. From our perspective, we consider later life as a period characterized by a multitude of stressors that individuals are likely to encounter. Each individual must confront a unique combination of stressors during this life stage, varying in their type, intensity and temporal occurrence. Measuring the overall level of this “late-life stress” is challenging. Nonetheless, even in the absence of explicit stressor exposure data, the present study provides meaningful insights into the factors that may foster or diminish the overall capacity for resilience in later life. Moreover, it paves the way for future research to delve into individuals’ responses to specific late-life challenges or stressors, and explore whether resilience to these could indeed be promoted by targeting the factors identified here (e.g., through experimental manipulations).

### Limitations and challenges for the network approach

Whereas network analysis is a valuable tool that can help to unravel the relative contributions of many factors, it is important to acknowledge its limitations. First, since our analyses relied on cross-sectional data, we cannot infer causality. We have discussed some plausible ideas as to how several risk and protective factors may influence individuals’ resilience capacity, and thereby affect quality of life and mental well-being. However, it is important to consider that some proposed pathways may be reversed or bi-directional, for example reducing worries/fears about death and dying, may also improve self-perceptions of aging; improving mental well-being, may also reduce depressive symptomatology. To determine the true intervening potential of these factors and uncover the underlying mechanisms of change, experimental manipulations are necessary. Second, network analysis does not account for the clinical relevance of the strength of the relationships. While being overshadowed by stronger relationships, certain weak associations may still be highly relevant for clinical practice. For instance, even though the link between perceived negative ageism and self-perceptions of aging may appear relatively weak compared to other observed relationships, it still makes a meaningful contribution to overall self-perceptions of aging, which in turn has strong implications for quality of life. Moreover, some factors may be weakly correlated with many other factors, leading to their role being ‘explained away’ in the partial correlation network. A case in point is ‘urbanization grade’, which showed strong bivariate relationships with many variables in the network, yet weak and negligible partial correlations in the network analysis. Such factors may not be detected as important predictors in this analysis, but could still have a substantial societal or clinical value. Hence, to fully comprehend the complex relationships among the variables, as well as their relevance to the field, it is important to consider current results in conjunction with other societal and clinical knowledge and/or use these insights to generate new hypotheses that should be tested in follow-up work in a confirmatory manner. Third, since we have no information about the evidence for the absence of edges, interpreting current findings, and particularly drawing inferences on the lack of certain relationships, warrants caution.

In addition to our main analyses, we explored potential moderating effects within the network. However, our moderated network analysis did not yield any significant three-way interactions, which seems most plausibly explained by the lack of statistical power. To gain a better understanding of the network dynamics, future research with a larger sample size is needed to uncover potential moderating effects.

### General limitations

A general limitation of the current study is the homogeneity of the sample. Participants were predominantly highly educated and had high household incomes. This may restrict the applicability of our results to people with lower educational attainment or different economic circumstances. Moreover, the requirement of technical proficiency with a computer, laptop or tablet may have introduced a bias. Individuals who are incapable of using these devises may differ in their characteristics and behaviors compared to those who are less technologically savvy or have limited access to such devices. Future research endeavors should aim to diversify the participant pool to encompass a broader socioeconomic spectrum and technological proficiency range (e.g., providing paper-pencil/offline alternatives), thereby enhancing the external validity of our findings and offering a more comprehensive understanding of the phenomena under investigation. Here, it may be particularly opportune to assess the moderating role of background, SES, and technological proficiency. Indeed, is has been suggested that individuals with a low socioeconomic position may rely more heavily on psychological and social resources that form the foundation of resilience capacity compared to their higher SES peers [184]. Consequently, the strength or nature of the interrelations among protective/risk factors and quality of life and mental well-being may vary as a function of SES.

In light of the significant variability among aging individuals and the timing of late-life challenges and transitions, we made the decision to refrain from directly controlling for age in our analysis. Incorporating age as a node within the network would yield limited informative value, as it seems unlikely to serve as a mediating factor. Nonetheless, investigating age as a potential moderator in future research with larger samples would be a valuable pursuit. This may shed light on the potential protective role of age, for example, due to increased self-awareness or the accumulation of life events that leads to an enlarged repertoire of knowledge and skills; [185]. Age may also have negative effect, for instance, due to a higher number of challenges.

Despite our comprehensive approach, the unexplained variance suggests that there are other factors beyond those considered in the present study that may play a significant role in shaping the resilience capacity among older adults. These factors could range from individual characteristics (e.g., nationality, culture, diet) and personality traits (e.g., extraversion, conscientiousness, self-control) to external circumstances and contextual factors (e.g., percentage of green space in the neighborhood, transport mobility) that were not taken into account. Adding such factors may change the structure of the network, resulting in a more nuanced understanding of the contributions of each of the factors.

## Conclusions

In sum, the findings of the present study offer new insights into the intricate web of resilience capacity-promoting (protective) and resilience capacity-reducing (risk) factors that may contribute to mental well-being and quality of life in later life, which we have interpreted in terms of a resilience-based framework of successful aging. Most prominently, we found a strong interconnected ‘psycho-social comfort community’, which overlapped strongly with a ‘positive outlook community’ and an ‘adaptive coping and social connectedness community’. Altogether, these communities emphasized the importance of self-efficacy, self-esteem, self-management ability and various other personality- and coping-related factors in nurturing both mental health and social connectedness and satisfaction in favor of mental well-being and quality of life. Moreover, they indicated the benefit of having a positive mindset. In addition, we demonstrated the importance of cultivating positive self-perceptions of aging and the potential benefits of targeting risk factors associated with negative self-perceptions for individuals’ quality of life in particular. Altogether, these findings shed light on the protective/ risk factors and domains that may have important implications for the process that enables mental well-being and quality of life at older age.

Our results have implications for clinical practice and society at large, as they may inform researchers and practitioners to design more effective interventions that foster resilience among older adults. The complex combination of inter-relations between risk and protective factors also underscores the importance of acknowledging the multi-dimensional nature of resilience and that individual differences in factors that impact the capacity for resilience may contribute to individual variations in how individuals age successfully. Moreover, this study elucidates why multi-pronged, holistic interventions, targeting multiple pathways simultaneously and allowing each person to derive specific benefits, may be especially promising to promote the overall capacity for resilience, and consequently quality of life and mental well-being among older adults. In addition to steering the development of more efficacious and personalized interventions, the insights gained from this study can also serve as a foundation for formulating other testable hypotheses and refining future longitudinal research in the field. Ultimately, by embracing this comprehensive approach, we can gain deeper insights and pave the way for tailored programs or societal campaigns that target the key factors crucial for fostering positive outcomes and for flourishing among older individuals as they navigate the journey of their later lives.

## Supporting information

S1 Appendix Supplementary materials. (PDF)

S2 Appendix Supplementary results. (PDF)
