# Supplementary material for: A cross-sectional network analysis of successful aging in a resilience-based framework
Source: PLoS One. 2025 Jan 15;20(1):e0315445. doi: 10.1371/journal.pone.0315445 (PMC11734968; doi:10.1371/journal.pone.0315445)
Supplement: S1 Appendix — (PDF) [file pone.0315445.s001.pdf]

Supporting Information [S1]: Supplementary materials

Supplement to: A cross-sectional network analysis of successful aging in a resilience-based framework

Demographics

Socio-economic status (SES)

Three different variables were used to determine one's socioeconomic status (SES): the subjective SES, the monthly net household income and the level of education. Cantril's 10-point self-anchoring ladder (Cantril, 1965) was used to assess the subjective SES. Participant were presented with a depiction of a ladder (steps numbered from one to ten) and are instructed to consider the ladder as a representation of where seniors in the Netherlands are standing, with seniors with the most money and education being at the top and those with the least money and education at the bottom. Subsequently, they were asked to indicate where they feel they stand on the ladder, resulting in a score ranging from 1 to 10 (SSES). Participants were asked to report on their net monthly household income by selecting one of the six categories used in the Tilburg Frailty Indicator (Gobbens et al., 2010; €600 or less to €2101 or more; INC). To assess the level of education, the Dutch Verhage (1964) scale was used. This scale consists of seven categories from unfinished primary education to university education (EDU). SSESS, INC and EDU scores were all recoded to 0 – 100, and averaged to obtain an overall SES score.

Urbanisation grade (URB)

Urbanisation grade was assessed by taking the mean number of addresses per km^2 within a circle radius of 500 m from the geographical centre (or centroid) of the six-digit postal code area of the participants (average size of such a postal code area is 0.0025 km^2). Data (from 2020) were retrieved from the national registration organisation Statistics Netherlands and pre-processed by the Geoscience and Health Cohort Consortium (GECCO).

(Mental) health

Happiness (HAP)

Happiness was measured using a single item question ('Do you feel happy in general?'), answered on a 11-point scale (0-10; Abdel-Khalek, 2006). Higher scores are indicative of greater happiness.

Mental well-being (MWB)

The 14-item Warwick Edinburgh Mental Wellbeing Scale (WEMWBS; Ikink et al., 2012; Tennant et al., 2007) was used to measure mental well-being (α = 0.89). All 14 items address positive aspects of mental health (e.g., "I've been feeling relaxed") and were scored on a 5-point Likert scale (1 = never, 2= barely, 3 = sometimes, 4 = often, 5 = always). Items were summed to a total of 14 to 70, with higher scores indicating better mental well-being.

Quality of Life (QoL)

The World Health Organization Quality of Life (WHOQOL)-OLD instrument (Gobbens & van Assen, 2016; Power et al., 2005) was used to assess QoL based on six subscales (four items each; α = 0.64 – 0.93): (1) sensory abilities (SAB; e.g., "To what extent do impairments to your senses (e.g., hearing, vision, taste, smell, touch) affect your daily life?"), (2) autonomy (AUT; e.g., "How much freedom do you have to make your own decisions?"), (3) past, present and future activities (PPF; e.g., "How satisfied are you with what you have achieved in life?"), (4) SOP (e.g., "To what extent do you feel that you have enough to do each day?"), (5) DAD (e.g., "How scared are you of dying?"), and (6) intimacy (INT; e.g., "To what extent do you feel a sense of companionship in your life?"). Responses were scored on 5-point Likert scales, with different wording, and summed across

each subscale (4 – 20), as well as to a total QoL score (24 – 120). Higher scores (per scale) indicate better quality of life. All items corresponding to the fifth subscale and some of items of the first and second subscale were reverse scored prior to summation.

Depression (DEP)

The 10-item Centre of Epidemiological Studies Short Depression Scale (CES-D-10; Andresen et al., 1994) was used to measure the level of depressive symptomatology ($\alpha = 0.82$). Each item (e.g., “I was bothered by things that usually don’t bother me”) was rated on a 4-point scale, ranging from 0 (less than one day) to 3 (5–7 days) and summed to a total score of 0 to 30. Prior to summation, two positively formulated items were reverse scored, such that higher scores were indicative of more depressive symptoms.

Anxiety (ANX)

Anxiety was measured using the 7-item anxiety subscale of the Hospital Anxiety and Depression Scale (HADS-A; Spinhoven et al., 1997; Zigmond & Snaith, 1983; $\alpha = 0.85$). Participants indicated to what extent they experienced feelings of restlessness, tenseness or panic over the past two weeks (e.g., “I’ve been feeling tensed lately”) on a Likert scale ranging from 0 (rarely or never) to 3 (mostly or always). Scores were summed to a total score ranging from 0 to 21, with higher scores indicating more anxiety symptoms.

Loneliness (LONE)

The 11-item Loneliness Scale was used to assess overall levels of loneliness (de Jong-Gierveld & van Tilburg, 1999; $\alpha = 0.87$). Six items are formulated negatively (emotional subscale; e.g., “I miss having a really close friend”) and 5 items positively (social subscale; e.g., “There is always someone I can talk to about my day-to-day problems). Possible answers are: “yes!”, “yes”, “more or less”, “no”, “no!”. Scores were calculated by counting the number of neutral and positive answers on the positively formulated items, and the number of neutral and negative answers on the negatively worded items. Higher scores reflected more loneliness (0-11).

Social-support discrepancy (SSD)

A shortened (12-item) version of the Social Support Discrepancy (SSD) subscale of the full Social Support List (SSL) was used to assess individuals’ satisfaction with the social support they receive (Kempen & van Eijk, 1995; van Eijk et al., 1994; van Sonderen, 1993; $\alpha = 0.90$). The full SSL combines measures of support interactions (SSL-I) and support satisfaction (SSL-D) in six subscales: (1) daily-oriented emotional support, (2) problem-oriented emotional support, (3) esteem, (4) instrumental support, (5) social companionship and (6) informative support. The SSL-I consists of 34 items that relate to all kinds of supporting interactions (e.g., receiving a compliment, being asked for help) and was created to assess the level of received social support, with higher scores reflecting more support. For the SSL-I, items are scored based on the following categories: 1 = barely or never, 2 = sometimes, 3 = regularly, 4 = very often. The SSL-D includes the same items, but the SSL-D asks individuals to rate the extent to which the obtained/received support meets one’s needs. Answer possibilities are: 1 = I miss it and I prefer more, 2 = I don’t really miss it, but I prefer more, 3 = exactly the right amount, 4 = it happens too often. Answers are recoded (option 3 and 4 = 1, option 2 = 2, and option 1 = 3), and summed to a total score, such that higher SSD scores indicate a larger perceived lack of support (12 – 36).

Health (HEA)

Health-related quality of life was assessed by the Short Form Health Survey (SF-36; Aaronson et al., 1998; Ware & Sherbourn, 1992; $\alpha = 0.78 - 0.87$). The SF-36 consists of several subscales, including (1) physical functioning (PF; “Does your health now limit you in [vigorous activities]?”, (2) role limitations due to physical (RP) or (3) emotional problems (RE; e.g., “during the past 4 weeks, have you had any problems with your work or other regular daily activities as a result of

your physical/emotional health?"), (4) energy/vitality (VT; e.g., "During the past 4 weeks, did you have a lot of energy?"), (5) emotional well-being (EW; e.g., "During the past 4 weeks, have you felt calm and peaceful?"), (6) social functioning (SF; e.g., "During the past 4 weeks, to what extent has your physical health or emotional problems interfered with your normal social activities with family, friends, neighbors, or groups?"), (7) pain (BP; e.g., "How much bodily pain have you had during the past 4 weeks?"), and (8) general health perception (GH; e.g., "In general, would you say your health is?"). Items were scored on different scales and recoded based on the guidelines of Van der Zee and Sanderman (2012). Both domain scores and a total SF-36 score were calculated, with higher scores reflecting better health (e.g., less pain, more energy, little limitations due to physical or emotional problems).

Physical/neurological diseases (PND)

To determine whether participants suffer from any physical and/or neurological disease, participants were asked to report whether they have a diagnosis for any of the following (chronic) conditions or diseases: (1) neurological condition (e.g., Parkinson's Disease or Multiple Sclerosis), (2) dizziness/imbalance, (3) rheumatic disease (e.g., osteoarthritis, osteoporosis), (4) Diabetes Mellitus, (5) lung disease (e.g., COPD, asthma), (6) cardiovascular disease (e.g., thrombosis, atherosclerosis), (7) cancer/tumor, (8) visual disturbance (e.g., cataracts, glaucoma), (9) consequences of a brain trauma (e.g., cerebral infarction, cerebral haemorrhage, severe concussion, whiplash), (10) consequences of cardiovascular problems, such as myocardial infarction or cardiac arrest, or (11) other. In case of multiple conditions or diseases, participants are instructed to select the condition or disease that has limited their wellbeing and functioning the most during de last couple of months. Participants can also indicate that they do suffer from any of the listed physical and/or neurological diseases, but that do not want to share any details with us.

Mental health disorders (MHD)

To determine whether participants suffer from any psychological or mental health problems, participants were asked to report whether a doctor or other health care professional has ever told them that they had a mental/psychological illness, disorder or health problem: (1) neurological developmental disorder (e.g., ADHD, autism), (2) abuse/addition of substances (e.g., alcohol, opioids or other drugs), (3) sleep disturbances (e.g., insomnia, narcolepsy), (4) post-traumatic stress disorder (PTSD), (5) stress-related problems (e.g., burnout, stress) , (6) other, for example: mood problems, eating disorder (e.g., anorexia nervosa, bulimia), obsessive-compulsive disorder, personality disorder (e.g., schizophrenia, psychosis). Again, in case of multiple illnesses, disorders or health problems, participants were instructed to select the condition or disease that has limited their wellbeing and functioning the most during de last couple of months. We also provide the option to indicate that they do suffer from any of the listed psychological or mental health problem, but that one chooses to keep the details of the type of condition to themselves.

Boredom (BORE)

To assess the extent to which someone is generally bored (i.e., feeling lethargic, listless, unmotivated and washed out, and not fascinated by anything, making it difficult to have fun), participants were asked to report to what extent these feelings apply to them using a 5-point Likert Scale (1= never, 2 = barely, 3 = sometimes, 4 = often, 5 = very often).

Coping/personality

Ability to bounce back (BRS)

The Brief Resilience Scale (BRS) was used to assess one's subjective ability to bounce back or recover from stress (Leontjevas et al., 2014; B. W. Smith et al., 2008; α = 0.89). The BRS consist of 3 positively (e.g., "I tend to bounce back quickly after hard times") and 3 negatively worded items (e.g., "I have a hard time making it through stressful events"), which are all scored on a 5-point Likert scale (1= strongly disagree, 2 = disagree, 3 = neutral, 4 = agree, 5 = strongly agree). Items are summed to a total ranging from 6 to 30, with higher scores reflecting higher levels of one's ability to bounce back or recover from stress. Negatively worded items are reverse coded prior to summation.

Positive appraisal (PAS)
The 14-item Positive Appraisal Style Scale (PASS; Veer et al., 2021) was used to assess positive appraisal content and processes, indicative for one's positive appraisal style (α = 0.86). The scale includes 10 items from the CERQ-short (Garnefski & Kraaij, 2006; e.g., "I think that I have to accept that this has happened"), covering 5 subscales (of two items): (1) positive reappraisal, (2) acceptance, (3) putting into perspective, (4) refocus on planning, and (5) positive refocusing, two self-generated items on (6) distanced stressor appraisal, and two items from the brief COPE (Carver, 1997), covering the (7) humor subscale. All items were scored on a 5-point Likert scale from (almost) never (1) to (almost) always (5), except for the two items of the humor subscale. These items were scored on a 4-point Likert scale (1= not at all, 2 = a little bit, 3 = quite a lot, 4 = a lot). A final PASS score was determined by taking the average of the z-normalized scores of all 14 items.

Behavioral coping (BC)
The 8-item Behavioral Coping Scale (BCS; Veer et al., 2021) was used to measure the extent to which individuals use behavioral coping strategies to deal with adversity (α = 0.79). Items of the BCS are derived from the brief COPE (Carver, 1997; e.g., "I've been getting emotional support from others"), and cover the following subscales: (1) use instrumental support, (2) seeking emotional support, (3) venting of emotions and (4) acting out (2 items per subscale). All items were scored on a 4-point Likert scale (1= not at all, 2 = a little bit, 3 = quite a lot, 4 = a lot) and summed to a total BC score ranging from 8 to 32.

General self-efficacy (GSE)
The General self-efficacy scale was used to assess how someone generally copes with stressors or challenging situations (Schwarzer & Jerusalem, 1995; Teeuw et al., 1994; α = 0.88). The scale concerns ten statements that ask how people generally think and act, focussing explicitly on a person's self-confidence that his or her actions are responsible for successful outcomes or that they have control over challenging demands in the environment (e.g., "I can solve most problems if I make the effort"). Each statement is scored on a 4-point Likert Scale (1 = not true at all, 2 = hardly true, 3 = moderately true, 4 = exactly true) and summed to a total score of 10 to 40. A higher score reflects higher self-efficacy.

Self-esteem (SE)
The Rosenberg self-esteem scale was used to assess global self-esteem (Franck et al., 2008; Rosenberg, 1979; α = 0.85). The scale includes 10 items (e.g., "On the whole, I am satisfied with myself") that are scored on a 4-point Likert scale (1 = totally disagree, 2 = disagree, 3 = agree, 4 = totally agree). After reverse coding some of the items (2, 5, 6, 8 and 9), items were summed up to a total of 10 to 40, with higher scores indicating a higher global self-esteem.

Self-management abilities (SMA)
The Self-Management Ability Scale (SMAS)-18 was used to measure self-management ability (Cramm, Strating, et al., 2012; α = 0.59 - 0.81). The 18-item version of the original 30-item SMAS (Schuurmans et al., 2005) consists of six 3-item subscales. Items corresponding to the taking

initiative (INI; e.g., “How often do you take initiative to get in touch with people who are dear to you?”), investing (INV; e.g., “Do you ensure that you have enough interests on a regular basis [such as a hobby] to keep you active?”), and positive frame of mind (PFM; e.g., “When you have a bad day, how often do you think that things will be better tomorrow?”) subscales are scored on a 6-point Likert scale ranging from never (1) to very often (6). Another 6-point scale, with 1=none, 2=one, 3=two, 4=three or four, 5=five or six, and 6=more than six, is used to score the items corresponding to variety subscale (VAR; e.g., “How many hobbies or activities do you have on a regular basis?”). Items corresponding to the multifunctionality subscale (MUL; e.g., “The activities I enjoy, I do together with others.”) are scored on a 5-point Likert scale ranging from strongly disagree (1) to strongly agree (5). Finally, items corresponding to the self-efficacy subscale (SEF; e.g., “Are you able to have friendly contacts with others?”) are scored on a 5-point Likert scale ranging from I am certain that I cannot (0) to I am completely certain that I can (5). Scores are calculated by recoding the scores to 0–5 or 0–4 (for the 6 and 5-point scales, respectively) and multiplying the items with six options by 4 and the items with five options by 5. After that, subscale scores are determined by taking the average of all items corresponding to each scale and multiplying that score by 5. Hence, subscale scores range from 0 to 100, with higher scores reflecting higher SMA in that dimension. SMAS total scores are calculated by taking the average of all mean subscale scores. Here, higher scores indicate a higher overall self-management ability.

Stress

Perceived Stress (PS)

The perception of stress during the last two weeks was assessed with the Perceived Stress Scale [79,80] (α = 0.78). The scale consists of 6 negatively (e.g., “In the last two weeks, how often have you felt nervous and “stressed”?”) and 4 positively (e.g., “in the last two weeks, how often have you felt that things were going your way?”) worded items, which were all scored on a 5-point Likert scale ranging from 1 (never) to 5 (very often). Scores were summed (positively worded items were reverse coded) across all scale items to a total of 0 to 40 points. Higher PS scores indicated higher levels of perceived stress.

Major Life Events (MLE)

We created a list of 18 life events/stressors to assess whether participants had experienced certain events or stressors throughout the last year (α = 0.59). Six of the items were based on the Tilburg Frailty Indicator (part A; Gobbens et al., 2010), and the other 12 items were selected from other life-event lists (e.g., Holmes and Rahe Stress Scale, 1967). The selection of these items was based on importance, suitability and potential impact for older adults. We also included some events that were not necessarily negative, but potential still burdensome (e.g., marriage, birth grandchild, retirement). Participants were asked whether a each event had happened in the past year, as well as to report how burdensome the event/stressor was to them (cf. Veer et al., 2021). Answer possibilities were 0 = this event did not happen, 1 = not burdensome at all, 2 = barely burdensome, 3 = somewhat burdensome, 4 = burdensome, 5 = very burdensome. A MLE major was calculated by summing the items scores to a total of 0 to 90, with higher scores indicating a larger burden of major life events in the past year.

Lifestyle

Sleep quality (SQ)

The Pittsburgh Sleep Quality Index is used to measure sleep quality of the past month (Buysse et al., 1989). The index consists of 19 questions, resulting in 7 component scores (ranging from 0: no difficulty to 3: severe difficulty) and one global sleep quality score (0 to 21), and can be used to estimate the severity of sleeping problems on the basis of the subjective representation of the participants. Higher scores indicate *worse/reduced sleep quality*. The component are: (1) subjective sleep quality (SSQ; i.e., ‘How would you rate your sleep quality overall?’), (2) sleep

latency (SL; i.e., time it takes to fall asleep), (3) sleep duration (SDUR), (4) habitual sleep efficiency (HSE; i.e., the percentage of time in bed that one is actually asleep), (5) sleep disturbances (SDIS; e.g., have to get up to use the bathroom), (6) use of sleep medication (SMED), and (7) daytime dysfunction (DAYDIS; e.g., 'How often have you had trouble staying awake while driving, eating meals or engaging in social activity?'). Scoring is based on specific instructions for each component.[1]

Physical activity (PHY)

Physical activity is assessed by using a limited number of questions, inspired by the Physical Activity Questionnaire used in the Longitudinal Aging Study Amsterdam (LASA team, 2020). First, participants are asked to indicate whether they are sitting in a wheelchair or not (WC). Next, they are asked to report the duration of their light to moderate physical activities, such as walking, cycling, and light household chores, in the past week (PA1). Similarly, they are asked to report the duration of their vigorous physical activities, such as intense bike riding, gymnastics, heavy household chores, etc., in the past week (PA2). An intensity-weighted total physical activity score is calculated by using Metabolic Equivalent of Task (MET) scores (one MET unit reflects 1kcal per kg body weight per hour). Normally, each activity is linked to a specific MET score, where the energy consumption of light to moderate physical activities ranges from 1.6 to 5.9 METs, and the energy consumption of vigorous physical activity is 6.0 MET or more. Here, we use an average MET score of 3.75 for mild to moderate activities, and 6.0 for vigorous activities. These scores are multiplied by the durations of the two intensity categories (PA1_MET for light to moderate, and PA2_MET for vigorous) and summed (PA). The resulting PHY score is adjusted based on whether the participant is sitting in a wheelchair or not (PA*WC). A score of 1 is assigned to those who are not in a wheelchair, while those in a normal wheelchair are assigned a score of 0.8, and those in a mechanical wheelchair are assigned a score of 0.5.

Alcohol Use (AU)

The Alcohol Use Disorders Identification Test was used to assess alcohol consumption and problems (Babor et al., 2001). The 10-item AUDIT has been developed to identify preliminary signs of hazardous drinking and alcohol use disorders and is regarded as the gold standard screening instrument in the adult population. All items are scored on a scale from 0 to 4, with slightly different category labels for each item. Lower item scores always indicate less (problematic) drinking. The first three items are used to detect hazardous drinking patterns, and measure the amount and frequency of alcohol consumption (Bush et al., 1998; Dawson et al., 2005; Saunders et al., 1993). The remaining seven questions, focusing on the frequency of experienced mental and physical problems due to drinking alcohol, are only asked when participants reach a predefined cut-off sum score on the first three items: 4 for men and 3 for woman (Reinert & Allen, 2007; Saunders et al., 1993). In the current study, only the sum score of the first three items is used to ensure scores among individuals are comparable.

Prospective and retrospective memory (PRM)

The Prospective and Retrospective Memory Questionnaire was used to measure prospective and retrospective memory failures in everyday life (Crawford et al., 2003; Gondo et al., 2010; G. Smith et al., 2000; Zimprich et al., 2011; α = 0.79). The questionnaire includes sixteen items, with half of the items concerning prospective memory failures (e.g., "Did you decide to do something in a few minutes' time and then forget to do it?"), and the other half of the items concerning retrospective memory failures (e.g., "Did you fail to recognise a place you have visited before?"). Participants are asked to indicate how often each of these failures generally happen on a 5-point Likert scale

[1] https://www.goodmedicine.org.uk/files/assessment,%20pittsburgh%20psqi.pdf

(1 = very often, 2 = quite often, 3 = sometimes, 4 = rarely, 5 = never), resulting in a total sum score of 8 to 40 for each memory construct (PM and RM), and a total PRM score of 16 to 80.

Contacts (CON)

To determine the extent to which one has meaningful social contacts, several questions were asked. Firstly, participants were asked to report to which extent they are satisfied with the social contacts and interactions one has on a monthly basis (SCON). Answers were given on a 5-point Likert Scale from very unsatisfied (1) to very satisfied (5). Secondly, participants had to indicate how many days per month (0 to 31) one has had face-to-face contact with someone outside their household (e.g., family, friends, neighbours, etc; RCON). Similarly, participants were asked to report how many days per month one has had contact with someone outside their household in an alternative way (e.g., calling, texting, e-mail, etc; ACON). A contacts composite score was calculated by adding the RCON and ACON scores, and multiplying this sum score to SCON.

Ageism

Negative self-perceptions of aging (SPoA)

The shortened version of the Aging Perceptions Questionnaire (APQ-S) was used to assess older adults' perceptions of aging (Slotman et al., 2015; α = 0.68 - 0.82). This scale consists of 21 items and comprises seven subscales. The chronic timeline (APQ1/TCr) subscale encompasses perceptions of aging that are chronic and constantly present (e.g., "I am aware of my age"), and the cyclical timeline (APQ2/TCy) subscale refers to perceptions that fluctuate (e.g., "I go through phases of feeling old"). The positive consequences (APQ3/CP) and negative consequences (APQ4/CN) subscales concern beliefs about the impact of aging on various life domains, either positive (e.g., "As I get older, I get wiser") or negative (e.g., "Getting older restricts the things that I can do"). The *emotional representations* (APQ5/ER) subscale refers to negative emotional responses to aging (e.g., "I get depressed when I think about getting older"). Finally, two subscales relate to beliefs about the extent to which one has control over various aspects of aging, both positive (APQ6/PCo, e.g., "The quality of my social life in later years depends on me") or negative (APQ7/NCo, e.g., "Slowing down with age is not something I can control") Answers are given on a 5-point scale, ranging from strongly disagree (1) to strongly agree (5), and items are summed to subscale scores, ranging from 3–15.To obtain an overall negative self-perceptions of aging measure (APQ/SpoA), the subscale scores of the positive dimensions (i.e., APQ3, APQ6) are reverse scored, and all seven subscale scores are averaged (21 – 105).

Perceived Negative Ageism (PNA) and Perceived Positive Ageism (PPA)

The 8-item Perceived Ageism Questionnaire was used to assess individuals' level of perceived negative (PNA; α = 0.80) and perceived positive (PPA; α = 0.74) ageism (Brinkhof et al., 2022). The PAQ-8 includes five items that reflect negative forms of ageism (PNA subscale) and three items that reflect positive forms of ageism (PPA subscale). Each of the items describe different situations or attitudes that older adults may have experienced or encountered in the past year. Participants were asked to report how often each situation has occurred, using a 5-point Likert scale (1 = never, 2 = barely, 3 = sometimes, 4 = often, 5 = very often). PNA and PPA subscales were calculated by taking the sum score of their corresponding items, ranging from 5 – 25 (PNA) and 3 – 15 (PPA).

COVID

Stringency Index

The stringency Index of the Oxford COVID-19: Government Response Tracker (OxCGRT)[2] was used to assess the strictness of 'lockdown style' policies that primarily restrict people's

[2] https://www.bsg.ox.ac.uk/research/research-projects/coronavirus-government-response-tracker

behaviour in the Netherlands at the time of participation. This index is a composite measure based on eight containment (i.e., closings of schools and universities, closings of workplaces, cancellations of public events, limits on private gatherings, closing of public transport, order to confine to the home, restrictions on internal movement between cities/regions, restrictions on international travel) and closure policies indicators and one health system policies indicator (i.e., presence of public info campaigns). If the inventory was not finished on the same day, but participation was divided over multiple days, the average stringency index of those days was used.

References

Aaronson, N. K., Muller, M., Cohen, P. D. A., Essink-Bot, M. L., Fekkes, M., Sanderman, R., Sprangers, M. A. G., Te Velde, A., & Verrips, E. (1998). Translation, validation, and norming of the Dutch language version of the SF-36 Health Survey in community and chronic disease populations. *Journal of Clinical Epidemiology*, *51*(11), 1055–1068. https://doi.org/10.1016/S0895-4356(98)00097-3

Abdel-Khalek, A. M. (2006). Measuring happiness with a single-item scale. In *Social Behavior and Personality* (Vol. 34, Issue 2, pp. 139–150). https://doi.org/10.2224/sbp.2006.34.2.139

Andresen, E. M., Malmgren, J. A., Carter, W. B., & Patrick, D. L. (1994). Screening for depression in well older adults: Evaluation of a short form of the CES-D. *American Journal of Preventive Medicine*, *10*(2), 77–84. https://doi.org/10.1016/s0749-3797(18)30622-6

Babor, T. F., Higgins-Biddle, J. C., Saunders, J. B., Monteiro, M. G., & World Health Organization. (2001). *AUDIT: the Alcohol Use Disorders Identification Test : Guidelines for Use in Primary Care, 2nd ed*. https://apps.who.int/iris/bitstream/handle/10665/67205/W?sequence=1

Brinkhof, L. P., de Wit, S., Murre, J. M. J., Krugers, H. J., & Ridderinkhof, K. R. (2022). The Subjective Experience of Ageism: The Perceived Ageism Questionnaire (PAQ). *International Journal of Environmental Research and Public Health*, *19*(14), 8792. https://doi.org/10.3390/ijerph19148792

Bush, K., Kivlahan, D. R., McDonell, M. B., Fihn, S. D., & Bradley, K. A. (1998). The AUDIT alcohol consumption questions (AUDIT-C): An effective brief screening test for problem drinking. *Archives of Internal Medicine*, *158*(16), 1789–1795. https://doi.org/10.1001/archinte.158.16.1789

Buysse, D. J., Reynolds, C. F., Monk, T. H., Berman, S. R., & Kupfer, D. J. (1989). The Pittsburgh sleep quality index: A new instrument for psychiatric practice and research. *Psychiatry Research*, *28*(2), 193–213. https://doi.org/10.1016/0165-1781(89)90047-4

Cantril, H. (1965). *Pattern of human concerns*.

Carver, C. S. (1997). You want to measure coping but your protocol’s too long: consider the brief COPE. *International Journal of Behavioral Medicine*, *4*(1), 92–100. https://doi.org/10.1207/s15327558ijbm0401_6

Cramm, J. M., Strating, M. M. H., de Vreede, P. L., Steverink, N., & Nieboer, A. P. (2012). Validation of the self-management ability scale (SMAS) and development and validation of a shorter scale (SMAS-S) among older patients shortly after hospitalisation. *Health and Quality of Life Outcomes*, *10*. https://doi.org/10.1186/1477-7525-10-9

Crawford, J. R., Smith, G., Maylor, E. A., Della Sala, S., & Logie, R. H. (2003). The Prospective and

Retrospective Memory Questionnaire (PRMQ): Normative data and latent structure in a large non-clinical sample. *Memory*, *11*(3), 261–275. https://doi.org/10.1080/09658210244000027

Dawson, D. A., Grant, B. F., Stinson, F. S., & Zhou, Y. (2005). Effectiveness of the derived Alcohol Use Disorders Identification Test (AUDIT-C) in screening for alcohol use disorders and risk drinking in the US general population. *Alcoholism: Clinical and Experimental Research*, *29*(5), 844–854. https://doi.org/10.1097/01.ALC.0000164374.32229.A2

De Jong-Gierveld, J., & Van Tilburg, T. (1999). Manual of the Loneliness Scale. *Fsw.vu.Nl*, 1–26. http://home.fsw.vu.nl/TG.van.Tilburg/manual_loneliness_scale_1999.html

Franck, E., De Raedt, R., Barbez, C., & Rosseel, Y. (2008). Psychometric properties of the Dutch Rosenberg self-esteem scale. *Psychologica Belgica*, *48*(1), 25–35. https://doi.org/10.5334/pb-48-1-25

Garnefski, N., & Kraaij, V. (2006). Cognitive emotion regulation questionnaire - development of a short 18-item version (CERQ-short). *Personality and Individual Differences*, *41*(6), 1045–1053. https://doi.org/10.1016/j.paid.2006.04.010

Gobbens, R. J. J., & van Assen, M. A. L. M. (2016). Psychometric properties of the Dutch WHOQOL-OLD. *Health and Quality of Life Outcomes*, *14*(1), 1–9. https://doi.org/10.1186/s12955-016-0508-5

Gobbens, R. J. J., van Assen, M. A. L. M., Luijkx, K. G., Wijnen-Sponselee, M. T., & Schols, J. M. G. A. (2010). The tilburg frailty indicator: Psychometric properties. *Journal of the American Medical Directors Association*, *11*(5), 344–355. https://doi.org/10.1016/j.jamda.2009.11.003

Gondo, Y., Renge, N., Ishioka, Y., Kurokawa, I., Ueno, D., & Rendell, P. (2010). Reliability and validity of the Prospective and Retrospective Memory Questionnaire (PRMQ) in young and old people: A Japanese study. *Japanese Psychological Research*, *52*(3), 175–185. https://doi.org/10.1111/j.1468-5884.2010.00433.x

Holmes, T. H., & Rahe, R. H. (1967). The social readjustment rating scale. *Journal of Psychosomatic Research*, *11*(2), 213–218. https://doi.org/10.1016/0022-3999(67)90010-4

Ikink, J. G. M., Lamers, S. M., & Boliuer, J. M. (2012). *De Warwick-Edinburgh Mental Well-being Scale (WEMWBS) als meetinstrument voor mentaal welbevinden in Nederland*. (Issue november).

Kempen, G. I. J. M., & Van Eijk, L. M. (1995). The psychometric properties of the SSL12-I, a short scale for measuring social support in the elderly. *Social Indicators Research*, *35*(3), 303–312. https://doi.org/10.1007/BF01079163

LASA team. (2020). *Physical activity - Longitudinal Aging Study Amsterdam*. https://lasa-vu.nl/topics/physical-activity/

Leontjevas, R., de Beek, W. O., Lataster, J., & Jacobs, N. (2014). Resilience to affective disorders: A comparative validation of two resilience scales. *Journal of Affective Disorders*, *168*, 262–268. https://doi.org/10.1016/j.jad.2014.07.010

Power, M., Quinn, K., & Schmidt, S. (2005). Development of the WHOQOL-Old module. *Springer*, *14*(10), 2197–2214. https://doi.org/10.1007/s11136-005-7380-9

Reinert, D. F., & Allen, J. P. (2007). The alcohol use disorders identification test: An update of research findings. In *Alcoholism: Clinical and Experimental Research* (Vol. 31, Issue 2, pp.

185–199). https://doi.org/10.1111/j.1530-0277.2006.00295.x

Rosenberg, M. (1979). *Conceiving the self.* New York: Basic Books. https://www.google.com/search?q=Rosenberg%2C+M.+(1979).+Conceiving+the+self.+New+York%3A+Basic+Books&rlz=1C1CHBD_nlNL904NL904&oq=Rosenberg%2C+M.+(1979).+Conceiving+the+self.+New+York%3A+Basic+Books&aqs=chrome..69i57.314j0j4&sourceid=chrome&ie=UTF-8

Saunders, J. B., AASLAND, O. G., BABOR, T. F., DE LA FUENTE, J. R., & GRANT, M. (1993). Development of the Alcohol Use Disorders Identification Test (AUDIT): WHO Collaborative Project on Early Detection of Persons with Harmful Alcohol Consumption-II. *Addiction*, *88*(6), 791–804. https://doi.org/10.1111/j.1360-0443.1993.tb02093.x

Schwarzer, R., & Jerusalem, M. (1995). General Self--efficacy Scale (GES). In *diabetes-psychologie.de*. https://diabetes-psychologie.de/downloads/Beschreibung_GSE.pdf

Slotman, A., Cramm, J. M., & Nieboer, A. P. (2015). Validation of the Dutch aging perceptions questionnaire and development of a short version. *Health and Quality of Life Outcomes*, *13*(1). https://doi.org/10.1186/s12955-015-0248-y

Smith, B. W., Dalen, J., Wiggins, K., Tooley, E., Christopher, P., & Bernard, J. (2008). The Brief Resilience Scale: Assessing the Ability to Bounce Back. *International Journal of Behavioral Medicine*, *15*(3), 194–200. https://doi.org/10.1080/10705500802222972

Smith, G., Sala, D., Logie, R. H., & Maylor, E. A. (2000). Prospective and retrospective memory in normal ageing and dementia: A questionnaire study. *Taylor & Francis*, *8*(5), 311–321. https://doi.org/10.1080/09658210050117735

Spinhoven, P., Ormel, J., Sloekers, P. P. A., Kempen, G. I. J. M., Speckens, A. E. M., & Van Hemert, A. M. (1997). A validation study of the hospital anxiety and depression scale (HADS) in different groups of Dutch subjects. *Psychological Medicine*, *27*(2), 363–370. https://doi.org/10.1017/S0033291796004382

Teeuw, B., Schwarzer, R., & Jerusalem, M. (1994). Dutch adaptation of the general self-efficacy scale. *Berlin, Germany*. https://meetinstrumentenzorg.nl/wp-content/uploads/instrumenten/328_3.pdf

Tennant, R., Hiller, L., Fishwick, R., Platt, S., Joseph, S., Weich, S., Parkinson, J., Secker, J., & Stewart-Brown, S. (2007). The Warwick-Edinburgh mental well-being scale (WEMWBS): Development and UK validation. *Health and Quality of Life Outcomes*, *5*(1), 63. https://doi.org/10.1186/1477-7525-5-63

Van der Zee, K. I., & Sanderman, R. (2012). *Het meten van de algemene gezondheidstoestand met de RAND-36, een handleiding. Tweede herziene druk*.

Van Eijk, L. M., Kempen, G. I., & Van Sonderen, F. L. (1994). A short scale for measuring social support in the elderly: the SSL12-I. *Tijdschrift Voor Gerontologie En Geriatrie*, *25*(5), 192.

Van Sonderen, E. (1993). Sociale steun lijst-Interacties (SSL-i) en Sociale steun lijst-Discrepanties (SSL-d). *Noordelijk Centrum Voor Gezondheidsvraagstukken*.

Veer, I. M., Riepenhausen, A., Zerban, M., Wackerhagen, C., Puhlmann, L. M. C., Engen, H., Köber, G., Bögemann, S. A., Weermeijer, J., Uściłko, A., Mor, N., Marciniak, M. A., Askelund, A. D., Al-Kamel, A., Ayash, S., Barsuola, G., Bartkute-Norkuniene, V., Battaglia, S., Bobko, Y., ... Kalisch, R. (2021). Psycho-social factors associated with mental resilience in the Corona lockdown. *Translational Psychiatry*, *11*(1), 1–11. https://doi.org/10.1038/s41398-020-01150-4

Verhage, F. (1964). *Intelligentie en leeftijd: Onderzoek bij Nederlanders van twaalf tot zevenenzeventig jaar. [Intelligence and Age: Investigations on Dutch Persons from Twelve to Seventy-Seven Years]* [Koninklijke van Gorcum]. https://research.rug.nl/en/publications/intelligentie-en-leeftijd-bij-volwassenen-en-bejaarden

Ware, J. E., & Sherbourn, C. D. (1992). The MOS 36-Item Short-Form Health Survey (SF-36): I . Conceptual Framework and Item Selection. *Medical Care*, *30*(6), 473–483.

Zigmond, A. S., & Snaith, R. P. (1983). The Hospital Anxiety and Depression Scale. *Acta Psychiatrica Scandinavica*, *67*(6), 361–370. https://doi.org/10.1111/j.1600-0447.1983.tb09716.x

Zimprich, D., Kliegel, M., & Rast, P. (2011). The factorial structure and external validity of the prospective and retrospective memory questionnaire in older adults. *European Journal of Ageing*, *8*(1), 39–48. https://doi.org/10.1007/s10433-011-0174-8
