# Supplementary material for: A cross-sectional network analysis of successful aging in a resilience-based framework
Source: PLoS One. 2025 Jan 15;20(1):e0315445. doi: 10.1371/journal.pone.0315445 (PMC11734968; doi:10.1371/journal.pone.0315445)
Supplement: S2 Appendix — (PDF) [file pone.0315445.s002.pdf]

Supporting Information [S2]: Supplementary results

Supplement to: A cross-sectional network analysis of successful aging in a resilience-based framework

This supplement includes an overview of some additional results that support the findings discussed in the main text, or have not been reported in the main text at all, but were performed as pre-registered.

Supporting information for main analyses

The strengths of the relationships of the main network (Figure 1A, main text) is shown in Table 1 of S2 Appendix. The stability of the edge weights, assessed using non-parametric bootstrapping, was visually shown in the main network, with dashed lines depicting relationships that were non-zero between 85% and 90% of the bootstraps (i.e.. relatively stable). and unstable relationships (non-zero < 85%) not being shown at all (to improve visibility). Table 1 of S2 Appendix shows the specific bootstrapped edge weights, including the quantile interval around this estimate, as well as the probability of each edge being present (non-zero) in the bootstrapping analysis.

Table 2 and 3 of S2 Appendix include the estimated parameters of the two-way interactions between/with categorical variables of interest, describing their (non-linear) relationships. Table B2 shows the interactions of several continuous variables with the categorical variable ‘Physical or neurological diseases’ (eight categories), reflecting the probability of every category. For instance. more negative self-perceptions of aging (SPoA) are associated with an increased probability of lung-related problems, (cardio)vascular diseases or having (a history of) cancer. Similarly, Table 3 of S2 Appendix shows the interactions of two continuous variables with the categorical variable ‘Mental health disorders’ (three categories).

Supporting information for the exploratory local structure analysis

The strengths of the relationships of the separate communities are shown in Tables 4-7 of S2 Appendix. These tables also include the stability of the edge weights, as well as the quantile intervals around the bootstrapped average and the probability of each edge being present (non-zero) in the bootstrapping analysis.

Table 8 of S2 Appendix shows the interactions of several continuous variables with the categorical variable ‘Physical or neurological diseases’ for the red community specifically.

Additional analyses, not reported in main text

As pre-registered, we also examined Moderated Mixed Graphical Models (or Moderated Network Models; MNMs) to examine differences across networks of with favorable and less favorable characteristics (e.g., general self-efficacy; negative self-perception of aging; depression; Haslbeck et al., 2018). We used the *mgm* package to estimate these MNMs with a nodewise regression approach, allowing us to evaluate all moderation effects involving categorical or continuous variables. A positive moderation effect on a certain relationship (e.g., between QoL and SPoA) would indicate that if one increases the value of the moderator, the pairwise interaction between QoL and SPoA becomes stronger. Surprisingly, we did not find any moderation effect, suggesting that none of the partial-correlations in the network was moderated by any of the other factors included in the network. This seems most plausibly explained by a lack of statistical power.

Table 1. The bootstrap (B) and sample (S) mean of the partial correlation coefficients of the edges of the main network, as well as the quantile intervals around the bootstrap mean.

Variable pair		Mean		Quantile Interval		Probability
		B	S	lower	upper	
LON	SSD	0.37	0.38	0.34	0.41	1
ANX	PS	0.34	0.34	0.31	0.38	1
DEP	ANX	0.32	0.32	0.28	0.35	1
SMA	CON	0.26	0.26	0.23	0.29	1
HAP	MWB	0.19	0.19	0.14	0.23	1
BRS	GSE	0.19	0.2	0.16	0.23	1
PAS	SMA	0.19	0.19	0.15	0.22	1
BC	SMA	0.18	0.18	0.14	0.21	1
HEA	PND	0.18	0.17	0.14	0.23	1
DEP	SQ	0.16	0.17	0.13	0.2	1
PAS	GSE	0.16	0.16	0.12	0.2	1
HAP	QoL	0.14	0.15	0.1	0.18	1
MWB	GSE	0.14	0.15	0.1	0.18	1
BRS	PAS	0.14	0.15	0.1	0.18	1
PAS	BC	0.13	0.13	0.09	0.17	1
GSE	SE	0.13	0.13	0.09	0.17	1
MWB	SE	0.12	0.13	0.09	0.16	1
SES	AU	0.11	0.1	0.07	0.14	1
MWB	BRS	0.11	0.12	0.08	0.15	1
PND	SPoA	0.11	0.09	0.07	0.16	0.99
SES	BC	0.1	0.11	0.06	0.13	1
MWB	QoL	0.1	0.1	0.07	0.14	1
MWB	SMA	0.1	0.11	0.07	0.14	1
SE	SMA	0.1	0.1	0.07	0.13	1
QoL	HEA	0.09	0.09	0.05	0.13	1
PNA	SPoA	0.09	0.07	0.05	0.13	0.98
DEP	PS	0.08	0.08	0.04	0.12	0.99
SMA	PHY	0.08	0.07	0.04	0.11	0.99
HEA	MHD	0.08	0.07	0.04	0.11	0.99
SQ	MHD	0.08	0.08	0.04	0.12	0.98
SES	HEA	0.07	0.07	0	0.11	0.92
PAS	PPA	0.07	0.06	0.03	0.11	0.95
SES	SPoA	0.06	0.07	0	0.12	0.78
MWB	PPA	0.06	0.07	0	0.1	0.78
QoL	SE	0.06	0.06	0	0.11	0.94
QoL	SMA	0.06	0.06	0	0.1	0.94
BRS	MHD	0.06	0.06	0	0.1	0.92
PS	MLE	0.06	0.07	0	0.11	0.8
PRM	PNA	0.06	0.05	0	0.1	0.89
PRM	SPoA	0.06	0.06	0	0.1	0.91
MWB	HEA	0.05	0.05	0	0.09	0.9
BRS	SE	0.05	0.05	0	0.08	0.91
PAS	PND	0.05	0.09	0	0.14	0.51
BC	SE	0.05	0.06	0	0.1	0.77
SMA	PPA	0.05	0.05	0	0.09	0.74
PND	PNA	0.05	0.05	0	0.1	0.81
QoL	PND	0.04	0.09	0	0.15	0.36
HEA	PHY	0.04	0.04	0	0.08	0.78
SES	QoL	0.03	0.06	0	0.09	0.53
SES	BRS	0.03	0.05	0	0.08	0.58
HAP	SE	0.03	0.03	0	0.07	0.61
BC	MHD	0.03	0.03	0	0.07	0.73
QoL	SSD	-0.02	0.03	-0.06	0	0.55
BRS	BC	-0.02	0.05	-0.08	0	0.26
SES	PNA	-0.03	0.03	-0.08	0	0.72
MWB	PS	-0.03	0.03	-0.07	0	0.7
PAS	SPoA	-0.03	0.04	-0.08	0	0.64
SE	PRM	-0.03	0.04	-0.08	0	0.55

SES	MLE	-0.04	0.04	-0.09	0	0.66
SE	BOR	-0.04	0.05	-0.09	0	0.8
SE	PNA	-0.04	0.03	-0.08	0	0.75
MWB	BOR	-0.05	0.07	-0.1	0	0.77
BRS	SPoA	-0.05	0.05	-0.09	0	0.9
PS	GSE	-0.05	0.06	-0.09	0	0.86
GSE	PRM	-0.05	0.05	-0.1	0	0.81
SMA	SPoA	-0.05	0.05	-0.09	0	0.89
QoL	BOR	-0.06	0.08	-0.11	0	0.76
QoL	PNA	-0.06	0.08	-0.11	0	0.83
PS	HEA	-0.06	0.06	-0.09	0	0.95
DEP	QoL	-0.08	0.08	-0.12	-0.05	1
ANX	HEA	-0.08	0.08	-0.12	-0.03	0.96
MLE	HEA	-0.08	0.09	-0.12	0	0.93
SE	SPoA	-0.08	0.08	-0.12	-0.05	1
SMA	BOR	-0.08	0.08	-0.12	-0.04	0.98
SSD	CON	-0.08	0.09	-0.12	-0.05	0.99
HAP	LON	-0.09	0.09	-0.13	-0.06	1
SMA	LON	-0.1	0.1	-0.14	-0.07	1
HEA	SPoA	-0.1	0.09	-0.13	-0.06	1
BC	LON	-0.12	0.13	-0.15	-0.09	1
PS	SE	-0.12	0.13	-0.17	-0.08	1
LON	CON	-0.12	0.13	-0.16	-0.09	1
HEA	SQ	-0.12	0.12	-0.16	-0.08	1
BRS	PS	-0.14	0.15	-0.18	-0.11	1
MWB	DEP	-0.16	0.16	-0.2	-0.12	1
DEP	HEA	-0.16	0.16	-0.2	-0.12	1
HAP	DEP	-0.19	0.19	-0.23	-0.15	1
QoL	LON	-0.2	0.2	-0.23	-0.17	1
QoL	SPoA	-0.27	0.27	-0.3	-0.23	1

The probability indicates how often a parameter was set to zero, based on 1000 bootstraps. Only the edges that were included in the main network are shown, including those who were considered unstable (non-zero < 85%) and therefore not shown in Figure 1 to improve visibility. The partial correlation coefficient of the categorial variables should also be interpreted with caution.

Table 2. Means, standard deviations and interactions of several continuous variables with each of the categories of the variable 'Physical or neurological diseases'.

		Negative SPoA		Health		Quality of Life		Positive appraisal style		Perceived negative ageism	
	n	*M (SD)*	*Probability*	*M (SD)*	*Probability*	*M (SD)*	*Probability*	*M (SD)*	*Probability*	*M (SD)*	*Probability*
1	392	53.6 (8.60)	0	77.9 (15.4)	-0.21	93.4 (9.34)	0	0.02 (0.60)	-0.12	7.79 (2.86)	0.04
2	71	54.4 (8.67)	0	81.4 (13.7)	0.22	91.0 (9.78)	-0.73	0.003 (0.59)	0	7.38 (2.79)	-0.16
3	82	54.4 (8.36)	**0.10**	80.0 (12.4)	0.08	93.1 (9.81)	0	-0.07 (0.64)	-0.30	8.04 (2.86)	**0.17**
4	39	59.0 (8.03)	**0.55**	70.6 (18.5)	-0.29	89.9 (8.96)	0.08	0.09 (0.56)	0.43	8.44 (2.58)	**0.19**
5	56	54.2 (8.77)	**0.14**	79.7 (16.5)	-0.21	95.0 (9.74)	0.21	0.11 (0.60)	0.17	7.38 (2.92)	0.00
6	76	52.9 (9.44)	-0.15	79.0 (10.9)	0.09	93.2 (8.32)	-0.03	0.09 (0.54)	0.11	7.41 (2.64)	-0.09
7	350	53.4 (9.32)	-0.13	77.6 (15.9)	-0.08	92.4 (10.4)	-0.19	0.06 (0.62)	0.04	7.87 (2.79)	0.07
8	934	49.5 (8.91)	-0.29	88.3 (8.54)	1.25	96.8 (8.65)	0.13	0.03 (0.59)	-0.20	6.94 (2.28)	-0.08

1 = Diabetes mellitus, dizziness, rheumatic disorders (e.g., arthrosis), consequences of, for example, a heart attack or cardiac arrest, consequences of a brain trauma (e.g., cerebral infarction, cerebral haemorrhage, severe concussion, whiplash), 2 = Visual impairment (e.g., cataracts or glaucoma), 3 = Cardiovascular disease (e.g.. thrombosis, atherosclerosis), 4 = Neurological disease (e.g., Parkinson's or MS) and others for which no details were given, 5 = Cancer/Tumor, 6 = Lung disease (e.g.. COPD or asthma), 7 = Any other disease not covered by the other categories, 8 = No. Parameters of the node-wise regressions represent the interactions of the continuous variables with each of the categories of the categorical variable, and reflect the probability of every category. Negative parameters depict that increases in the continuous variable are associated with decreases of the probability of that given category, and vice versa. For instance, more negative self-perceptions of aging (SPoA) are associated with an increased probability of (cardio)vascular or neurological diseases or having (a history of) cancer. Higher levels of perceived negative ageism are associated with an increased probability of (cardio)vascular or neurological diseases. These examples are shown in bold.

Table 3. Means. standard deviations and interactions of several continuous variables with each of the categories of the variable 'Mental Health Disorders'.

		**Reduced sleep quality**		**Health**		**Behavioral coping**		**Ability to bounce back or recover from stress**		**Mental well-being**	
	n	*M (SD)*	*Probability*	*M (SD)*	*Probability*	*M (SD)*	*Probability*	*M (SD)*	*Probability*	*M (SD)*	*Probability*
1	200	7.61 (4.08)	**0.18**	72.1 (17.9)	-0.04	21.7 (3.84)	0	19.2 (3.94)	0	52.4 (6.56)	0
2	199	6.01 (3.66)	0	77.9 (18.0)	0	22.1 (3.88)	0	19.5 (4.34)	0	53.0 (6.15)	0
3	1601	4.93 (2.90)	**-0.09**	84.8 (11.4)	0.24	21.5 (3.60)	-0.08	21.7 (3.66)	0.22	56.3 (5.52)	0.11

1 = Neurodevelopmental disorder (e.g.. ADHD. autism), substance abuse/dependence (e.g., alcohol, opioids. or other drugs), PTSD, or other problems not covered by the other categories, for instance mood problems, eating disorder, obsessive-compulsive disorder or personality disorder, 2 = Other stress-related problems (burn-out, overworked), 3 = No mental health problems/disorders. Parameters of the node-wise regressions represent the interactions of the continuous variables with each of the categories of the categorical variable, and reflect the probability of every category. Negative parameters depict that increases in the continuous variable are associated with decreases of the probability of that given category. and vice versa. For instance, elevated levels of reduced sleep quality (i.e.. more sleep problems) seem to be associated with a reduced probability of having no mental health problems/disorders, and increased probability of neurodevelopmental disorder, substance abuse/dependence, PTSD or other problem not covered by the other categories. These examples are shown in bold.

Table 4. The bootstrap (B) and sample (S) mean of the partial correlation coefficients of the edges of the yellow community network, as well as the quantile intervals around the bootstrap mean.

Variable pair		Mean		Quantile Interval		Probability
		B	S	lower	upper	
INI	INV	0.46	0.46	0.43	0.49	1
PPF	SOP	0.33	0.34	0.3	0.37	1
AUT	PPF	0.3	0.31	0.26	0.34	1
SSL	EMO	0.3	0.31	0.26	0.35	1
PSS	ANX	0.29	0.3	0.26	0.33	1
SOC	EMO	0.28	0.29	0.24	0.32	1
PF	BP	0.28	0.28	0.24	0.33	1
RP	SF	0.24	0.24	0.19	0.29	1
VT	EW	0.24	0.24	0.2	0.28	1
DEP	ANX	0.23	0.24	0.2	0.27	1
VAR	MUL	0.23	0.23	0.2	0.27	1
PF	GH	0.21	0.21	0.17	0.25	1
VT	GH	0.2	0.2	0.16	0.23	1
HS	EW	0.18	0.18	0.15	0.21	1
RP	BP	0.18	0.18	0.13	0.23	1
RE	EW	0.17	0.18	0.13	0.22	1
HS	INT	0.16	0.17	0.12	0.19	1
DEP	SQ	0.16	0.17	0.12	0.2	1
INV	SEF	0.16	0.16	0.12	0.19	1
PF	RP	0.16	0.16	0.11	0.21	1
RE	SF	0.16	0.15	0.11	0.21	1
HS	MWB	0.15	0.15	0.11	0.2	1
CON	INI	0.15	0.15	0.12	0.18	1
VT	SF	0.14	0.14	0.09	0.18	1
DAD	GH	0.13	0.14	0.1	0.16	1
INI	SEF	0.13	0.14	0.1	0.17	1
INV	VAR	0.13	0.13	0.09	0.17	1
SAB	GH	0.12	0.12	0.08	0.15	1
SF	BP	0.12	0.12	0.07	0.16	1
HS	PPF	0.11	0.11	0.07	0.15	1
MWB	PPF	0.11	0.11	0.07	0.14	1
MWB	VT	0.11	0.11	0.08	0.14	1
MWB	EW	0.11	0.12	0.08	0.15	1
SOP	INV	0.11	0.11	0.08	0.14	1
BP	GH	0.11	0.11	0.06	0.15	1
INI	MUL	0.1	0.1	0.06	0.14	1
MUL	SEF	0.1	0.1	0.06	0.14	1
PF	VT	0.1	0.09	0.05	0.15	0.98
SSL	SOC	0.09	0.1	0.05	0.13	1
VAR	SEF	0.09	0.1	0.06	0.13	1
RP	RE	0.09	0.08	0.03	0.15	0.96
RP	VT	0.09	0.09	0.04	0.13	0.99
MWB	SEF	0.08	0.08	0.05	0.11	1
INT	MUL	0.07	0.07	0.03	0.1	0.97
CON	VAR	0.07	0.07	0.04	0.11	1
CON	SEF	0.07	0.07	0.03	0.1	0.97
INV	MUL	0.07	0.07	0	0.11	0.94
RP	GH	0.07	0.07	0.02	0.11	0.97
AUT	GH	0.06	0.06	0	0.09	0.94
PPF	INT	0.06	0.07	0	0.1	0.94
PPF	SEF	0.06	0.07	0.03	0.1	0.99
DEP	PSS	0.05	0.06	0	0.09	0.92
DEP	EMO	0.05	0.06	0	0.08	0.94
SAB	SF	0.05	0.06	0	0.1	0.76
CON	MUL	0.05	0.05	0	0.09	0.94
INV	PFM	0.05	0.06	0	0.09	0.65
SEF	PFM	0.05	0.05	0	0.09	0.77

SF	GH	0.05	0.05	0	0.09	0.88
PPF	EW	0.04	0.04	0	0.07	0.79
PFM	EW	0.04	0.06	0	0.1	0.62
VT	BP	0.04	0.04	0	0.08	0.74
MWB	INV	0.03	0.04	0	0.08	0.55
PPF	GH	0.02	0.03	0	0.06	0.51
PSS	SAB	-0.01	0.03	-0.06	0	0.34
PSS	PPF	-0.01	0.02	-0.05	0	0.38
DEP	SF	-0.02	0.03	-0.06	0	0.44
PSS	AUT	-0.02	0.04	-0.07	0	0.48
PSS	SF	-0.02	0.03	-0.07	0	0.57
SSL	SEF	-0.02	0.03	-0.06	0	0.36
EMO	EW	-0.03	0.03	-0.06	0	0.61
PPF	SOC	-0.04	0.06	-0.08	0	0.78
SSL	RE	-0.04	0.05	-0.09	0	0.78
HS	EMO	-0.05	0.05	-0.08	0	0.83
DEP	SOP	-0.05	0.06	-0.09	0	0.79
DEP	RE	-0.05	0.06	-0.1	0	0.77
SQ	BP	-0.05	0.06	-0.09	0	0.89
CON	SOC	-0.06	0.06	-0.1	-0.02	0.95
CON	EMO	-0.06	0.07	-0.1	0	0.82
PPF	SSL	-0.07	0.08	-0.11	-0.02	0.95
MWB	PSS	-0.08	0.08	-0.12	-0.05	1
DEP	VT	-0.08	0.08	-0.12	-0.04	0.99
PSS	PFM	-0.08	0.09	-0.12	-0.04	0.96
SQ	VT	-0.08	0.09	-0.13	0	0.89
SEF	EMO	-0.08	0.08	-0.11	-0.04	0.96
CON	SSL	-0.09	0.1	-0.13	-0.06	1
HS	DEP	-0.12	0.12	-0.16	-0.08	1
MWB	DEP	-0.12	0.12	-0.16	-0.09	1
SEF	SOC	-0.13	0.13	-0.17	-0.1	1
SOP	EMO	-0.14	0.14	-0.17	-0.11	1
DEP	EW	-0.15	0.15	-0.19	-0.11	1
ANX	DAD	-0.15	0.16	-0.19	-0.11	1
PSS	EW	-0.16	0.15	-0.2	-0.11	1
INT	SOC	-0.18	0.18	-0.21	-0.14	1
INT	EMO	-0.19	0.2	-0.23	-0.15	1
ANX	EW	-0.25	0.25	-0.28	-0.21	1

The probability indicates how often a parameter was set to zero, based on 1000 bootstraps. Only the edges that were included in the yellow community network are shown, including those who were considered unstable (non-zero < 85%), or who had a partial correlation coefficient smaller than 0.05, and were therefore not shown in Figure 3 to improve visibility.

Table 5. The bootstrap (B) and sample (S) mean of the partial correlation coefficients of the edges of the red community network, as well as the quantile intervals around the bootstrap mean.

Variable pair		Mean		Quantile Interval		Probability
		B	S	lower	upper	
PPF	SOP	0.39	0.39	0.35	0.42	1
VT	EW	0.37	0.38	0.34	0.41	1
TCY	ER	0.32	0.32	0.28	0.35	1
AUT	PPF	0.31	0.31	0.27	0.34	1
PF	BP	0.26	0.26	0.22	0.31	1
RE	EW	0.26	0.27	0.22	0.3	1
TCY	CN	0.24	0.24	0.21	0.28	1
RP	SF	0.24	0.24	0.19	0.29	1
PND	GH	0.23	0.23	0.19	0.28	1
PPF	RSES	0.21	0.22	0.18	0.25	1
CN	NCO	0.2	0.2	0.16	0.24	1
EW	RSES	0.2	0.2	0.16	0.23	1
PCO	NCO	0.19	0.2	0.15	0.23	1
PPF	INT	0.19	0.19	0.15	0.22	1
VT	GH	0.19	0.19	0.16	0.22	1
RP	BP	0.18	0.18	0.13	0.23	1
TCR	CN	0.17	0.16	0.13	0.21	1
PF	RP	0.16	0.16	0.11	0.21	1
RE	SF	0.16	0.16	0.11	0.21	1
PND	PF	0.14	0.12	0.1	0.18	1
PF	GH	0.14	0.14	0.1	0.18	1
VT	SF	0.14	0.15	0.09	0.19	1
PND	BP	0.13	0.1	0.1	0.17	1
PPF	EW	0.12	0.12	0.08	0.15	1
SF	BP	0.12	0.12	0.07	0.16	1
CP	PCO	0.11	0.12	0.07	0.15	1
PF	VT	0.1	0.1	0.05	0.15	0.98
TCR	TCY	0.09	0.08	0.05	0.13	0.97
PND	SAB	0.09	0.08	0.06	0.13	0.98
DAD	GH	0.09	0.1	0.06	0.12	1
RP	RE	0.09	0.1	0.03	0.15	0.97
RP	VT	0.09	0.1	0.05	0.14	0.99
EW	SF	0.09	0.1	0	0.14	0.93
CN	PND	0.08	0.06	0	0.13	0.89
BP	GH	0.08	0.08	0.04	0.13	0.98
ER	PCO	0.07	0.07	0.03	0.1	0.96
ER	NCO	0.06	0.06	0	0.1	0.94
RP	GH	0.06	0.07	0.02	0.11	0.96
PNA	PND	0.05	0.06	0	0.1	0.68
SAB	SF	0.05	0.06	0	0.1	0.77
SAB	GH	0.05	0.05	0	0.09	0.84
SF	GH	0.05	0.05	0	0.09	0.91
AUT	GH	0.04	0.05	0	0.07	0.79
SOP	VT	0.04	0.06	0	0.08	0.74
SOP	EW	0.04	0.05	0	0.09	0.7
INT	EW	0.04	0.06	0	0.09	0.59
CP	CN	0.03	0.03	0	0.07	0.6
CP	NCO	0.03	0.04	0	0.07	0.64
CP	PND	0.03	0.08	0	0.11	0.29
CN	ER	0.03	0.03	0	0.08	0.7
DAD	EW	0.03	0.05	0	0.08	0.66
RE	VT	0.03	0.03	0	0.08	0.52
VT	BP	0.03	0.04	0	0.08	0.6
CP	SAB	-0.02	0.02	-0.06	0	0.41
NCO	GH	-0.02	0.02	-0.05	0	0.47

PF	EW	-0.02	0.03	-0.09	0	0.39
CN	SOP	-0.03	0.03	-0.07	0	0.61
PCO	GH	-0.03	0.03	-0.07	0	0.64
PNA	RSES	-0.05	0.05	-0.09	0	0.74
TCY	GH	-0.06	0.07	-0.1	-0.03	0.96
ER	RSES	-0.06	0.06	-0.1	-0.02	0.96
NCO	PF	-0.07	0.07	-0.1	-0.03	0.96
CP	RSES	-0.08	0.08	-0.12	-0.04	0.96
CN	PF	-0.08	0.08	-0.12	-0.05	0.99
PCO	AUT	-0.08	0.09	-0.12	-0.04	0.98
TCY	RSES	-0.09	0.09	-0.12	-0.05	1
CN	SAB	-0.09	0.09	-0.13	-0.06	0.99
ER	EW	-0.09	0.09	-0.12	-0.06	1
CN	GH	-0.1	0.11	-0.14	-0.07	1
PCO	PPF	-0.1	0.11	-0.15	-0.06	0.98
PNA	SAB	-0.14	0.14	-0.18	-0.1	1
ER	DAD	-0.3	0.31	-0.34	-0.26	1
NCO	GH	-0.02	0.02	-0.05	0	0.47
PF	EW	-0.02	0.03	-0.09	0	0.39
CN	SOP	-0.03	0.03	-0.07	0	0.61
PCO	GH	-0.03	0.03	-0.07	0	0.64

The probability indicates how often a parameter was set to zero, based on 1000 bootstraps. Only the edges that were included in the red community network are shown, including those who were considered unstable (non-zero < 85%), or who had a partial correlation coefficient smaller than 0.05, and were therefore not shown in Figure 3 to improve visibility.

Table 6. The bootstrap (B) and sample (S) mean of the partial correlation coefficients of the edges of the blue community network, as well as the quantile intervals around the bootstrap mean.

Variable pair		Mean		Quantile Interval		Probability
		**B**	**S**	**lower**	**upper**	
PAS	PFM	0.48	0.48	0.45	0.51	1
INI	INV	0.47	0.48	0.44	0.5	1
SOC	EMO	0.46	0.47	0.42	0.5	1
VAR	MUL	0.25	0.26	0.21	0.29	1
INV	SEF	0.18	0.19	0.15	0.22	1
BC	PAS	0.15	0.14	0.11	0.19	1
INI	SEF	0.15	0.15	0.12	0.19	1
INV	VAR	0.15	0.15	0.11	0.18	1
INI	MUL	0.12	0.13	0.08	0.16	1
VAR	SEF	0.12	0.12	0.08	0.15	1
MUL	SEF	0.12	0.12	0.09	0.16	1
BC	SEF	0.09	0.09	0.05	0.13	1
SEF	PFM	0.09	0.10	0.06	0.13	1
BC	INI	0.08	0.08	0.04	0.12	0.98
PAS	MUL	0.07	0.08	0.03	0.11	0.98
INV	MUL	0.07	0.08	0.03	0.11	0.98
BC	VAR	0.05	0.05	0	0.09	0.89
PAS	INV	0.04	0.04	0	0.07	0.79
INV	PFM	0.04	0.05	0	0.08	0.81
INI	VAR	0.03	0.03	0	0.07	0.56
INI	SOC	-0.03	-0.04	-0.07	0	0.73
PFM	EMO	-0.03	-0.04	-0.07	0	0.63
MUL	SOC	-0.04	-0.05	-0.08	0	0.81
SEF	SOC	-0.15	-0.15	-0.18	-0.11	1
SEF	EMO	-0.20	-0.21	-0.24	-0.17	1
BC	SOC	-0.21	-0.19	-0.25	-0.17	1

The probability indicates how often a parameter was set to zero, based on 1000 bootstraps. Only the edges that were included in the blue community network are shown, including those who were considered unstable (non-zero < 85%), or who had a partial correlation coefficient smaller than 0.05, and were therefore not shown in Figure 3 to improve visibility.

Table 7. The bootstrap (B) and sample (S) mean of the partial correlation coefficients of the edges of the navy community network, as well as the quantile intervals around the bootstrap mean.

Variable pair		Mean		Quantile Interval		Probability
		**B**	**S**	**lower**	**upper**	
MWB	SE	0.28	0.28	0.25	0.32	1
PASS	GSE	0.21	0.21	0.17	0.25	1
GSE	BRS	0.21	0.21	0.17	0.25	1
BRS	MWB	0.2	0.19	0.16	0.23	1
GSE	MWB	0.18	0.18	0.14	0.22	1
PAS	BRS	0.17	0.17	0.13	0.21	1
GSE	SE	0.16	0.16	0.13	0.2	1
PAS	MWB	0.08	0.08	0.04	0.12	0.98
PAS	SE	0.08	0.08	0.04	0.12	0.99
BRS	SE	0.08	0.08	0.04	0.12	1
GSE	PSS	-0.07	0.07	-0.11	-0.03	0.97
PSS	SE	-0.22	0.22	-0.26	-0.18	1
BRS	PSS	-0.24	0.24	-0.27	-0.2	1
MWB	PSS	-0.24	0.24	-0.28	-0.19	1

Note. The probability indicates how often a parameter was set to zero, based on 1000 bootstraps. Only the edges that were included in the navy community network are shown

Table 8. Means. standard deviations and interactions of several continuous variables with each of the categories of the variable 'Physical or neurological diseases' in the red community.

		**Sensory abilities**		**General health perception**		**Physical functioning**		**Bodily pain (-)**	
	n	*M (SD)*	*Probability*	*M (SD)*	*Probability*	*M (SD)*	*Probability*	*M (SD)*	*Probability*
1	392	17.3 (2.56)	0	62.0 (18.4)	0.17	80.1 (18.5)	0	73.4 (19.7)	-0.68
2	71	15.8 (2.94)	-0.56	65.6 (14.6)	0.34	86.1 (14.7)	0.19	84.3 (16.6)	-0.05
3	82	17.4 (2.31)	0.11	57.9 (16.5)	**-0.38**	80.4 (18.7)	-0.13	82.8 (16.7)	0.09
4	39	16.4 (3.15)	0	48.5 (20.9)	**-0.51**	67.9 (28.6)	-0.36	75.6 (23.1)	0
5	56	17.7 (2.61)	0.20	57.9 (20.1)	**-0.54**	83.7 (18.9)	0	83.8 (17.3)	0.13
6	76	17.3 (2.66)	-0.08	60.8 (16.1)	-0.17	82.7 (15.0)	-0.16	81.9 (14.8)	0
7	350	17.1 (3.03)	-0.10	63.2 (18.4)	0.31	83.9 (16.9)	0.28	76.6 (22.6)	-0.46
8	934	18.2 (2.23)	0.06	76.7 (13.5)	0.85	93.4 (8.79)	0.68	90.0 (12.4)	0

1 = Diabetes mellitus, dizziness, rheumatic disorders (e.g.. arthrosis), consequences of, for example, a heart attack or cardiac arrest, consequences of a brain trauma (e.g.. cerebral infarction, cerebral haemorrhage, severe concussion. whiplash), 2 = Visual impairment (e.g.. cataracts or glaucoma), 3 = Cardiovascular disease (e.g.. thrombosis, atherosclerosis), 4 = Neurological disease (e.g.. Parkinson's or MS) and others for which no details were given, 5 = Cancer/Tumor, 6 = Lung disease (e.g.. COPD or asthma), 7 = Any other disease not covered by the other categories, 8 = No. Parameters of the node-wise regressions represent the interactions of the continuous variables with each of the categories of the categorical variable. and reflect the probability of every category. Negative parameters depict that increases in the continuous variable are associated with decreases of the probability of that given category, and vice versa. For instance, high levels of general health perception are associated with a decreased probability of having a cardiovascular or neurological disease, as well as (a history) of cancer. These examples are shown in bold.
